# Novel non-coding FOXP3 transcript isoform associated to potential transcriptional interference in human regulatory T cells

**DOI:** 10.1080/15476286.2025.2502719

**Published:** 2025-07-02

**Authors:** Marcos Cases, Niclas Ritter, Hector Rincon-Arevalo, Sandy Kroh, Aysegül Adam, Marieluise Kirchner, Hanieh Moradian, Manfred Gossen, Maria Dzamukova, Artür Manukyan, Markus Landthaler, Christopher Kressler, Anja E. Hauser, Daniel P. Depledge, Julia K. Polansky, Hyun-Dong Chang

**Affiliations:** a Flow Cytometry Core Facility, Deutsches Rheuma-Forschungszentrum Berlin, A Leibniz Institute (DRFZ), Berlin, Germany; b Institute of Virology, Hannover Medical School, Hannover, Germany; c Department of Medicine, Rheumatology and Clinical Immunology, Charité - Universitätsmedizin Berlin, Berlin, Germany; d Deutsches Rheuma-Forschungszentrum Berlin, Ein Institut der Leibniz-Gemeinschaft, Berlin, Germany; e Grupo de Inmunología Celular e Inmunogenética GICIG, Universidad de Antioquia, Medellín, Colombia; f Division of Rheumatology and Clinical Immunology, Charité, Universitätsmedizin Berlin, Berlin, Germany; g Immune Dynamics, Deutsches Rheuma-Forschungszentrum (DRFZ), A Leibniz Institute, Berlin, Germany; h Department of Periodontology, Oral Medicine and Oral Surgery, Charité - Universitätsmedizin Berlin, Berlin, Germany; i Core Unit Proteomics, Berlin Institute of Health at Charité- Universitätsmedizin Berlin and Max Delbrück Center for Molecular Medicine (MDC), Berlin, Germany; j Institute of Active Polymers, Helmholtz-Zentrum Hereon, Teltow, Germany; k Berlin Institute of Health Center for Regenerative Therapies (BCRT), Berlin, Germany; l Berlin Institute for Medical Systems Biology (BIMSB), Max Delbrück Center for Molecular Medicine, Berlin, Germany; m Berlin Institute of Health (BIH) at Charité Universitätsmedizin Berlin, BIH Center for Regenerative Therapies (BCRT), Immuno-Epigenetics Group, Berlin, Germany; n German Center for Infection Research (DZIF), Partner Site Hannover-Braunschweig, Hannover, Germany; o Cluster of Excellence RESIST (EXC 2155), Hannover Medical School, Hannover, Germany; p Immuno-Epigenetics, Deutsches Rheuma-Forschungszentrum Berlin, A Leibniz Institute (DRFZ), Berlin, Germany; q Schwiete laboratory for microbiota and inflammation, DRFZ, Berlin, Germany; r Chair of Cytometry, Institute for Biotechnology, Technische Universtität Berlin, Berlin Germany

**Keywords:** FOXP3, T_REG_, alternative promoter, transcript isoform, transcriptional interference

## Abstract

CD4+ regulatory T cells (T_REGS_) are critical for immune tolerance and the transcription factor Forkhead Box P3 (FOXP3) plays a crucial role in their differentiation and function. Recently, an alternative promoter has been reported for FOXP3, which is active only in T_REGS_ and could have profound implications for the output of the locus, and therefore, for the functionality of these cells. By direct RNA sequencing we identified multiple novel FOXP3 transcriptional products, including one relatively abundant isoform with an extended 5’ UTR that we named ‘longFOXP3’. Western blotting, analysis of public mass spectrometry data, and transfection of *in vitro* transcribed RNA suggested that longFOXP3 is not coding. Furthermore, we show using two distinct RNA single-molecule fluorescence in situ hybridization technologies that transcription from the upstream promoter correlates with decreased levels of FOXP3 at the mRNA and protein levels. Together, we provide compelling evidence that the transcriptional output of the human FOXP3 locus is far more complex than that of the current annotation and warrants a more detailed analysis to identify coding and non-coding transcript isoforms. Furthermore, the alternative promoter may interfere with the activity of the canonical promoter, evoking intragenic transcriptional interference, and in this way, fine-tune the levels of FOXP3 in human T_REGS_.

## 1. Introduction

CD4+ regulatory T cells (T_REGS_) perform a critical role for immune homoeostasis and tolerance to both self and non-self [1]. The transcription factor (TF) Forkhead Box Protein 3 (FOXP3) is central to T_REG_ biology, as it is not only required for their differentiation, but also for their function [2]. The expression and function of FOXP3 itself is in turn subject to an intricate regulation that takes place at the epigenetic, transcriptional, and post-translational levels [3]. Recently, the gene has been reported to be also under direct negative regulation by FLICR, a lncRNA positioned in tandem upstream of FOXP3 (S1 Fig) [1].

Located on the X-chromosome and transcribing in a centromeric to telomeric orientation, the human FOXP3 gene comprises 12 exons. The exons are numbered from −1 to 11, with the complete open reading frame (ORF) contained in exons 1 to 11 (S1 Fig). In contrast to the mouse FOXP3 gene, which encodes a single protein, the human FOXP3 gene encodes two major proteoforms due to alternative splicing: a ‘full-length’ variant (FOXP3_fl) and an isoform in which the amino acid sequence encoded by exon 2 is missing (FOXP3_∆2) [4]. Although initially considered to contribute equally to T_REG_ differentiation and function, it was recently reported that patients who only express the FOXP3_Δ2 isoform suffer from immune dysregulation, polyendocrinopathy, enteropathy, X-linked (IPEX) syndrome, and that FOXP3_fl plays a crucial role in T_REG_ stability and in immune homoeostasis [4].

As clearly exemplified above, an increase in transcriptome diversity can have a profound impact on cell physiology because changes in the ORF of mRNA molecules can change the identity and functionality of encoded proteins, and extension or shortening of the transcript’s UTR can affect post-transcriptional gene regulation [5]. In addition to alternative splicing, alternative promoter usage can increase transcriptome complexity [6–8]. Early attempts to annotate the human genome in the first decade of the 21^st^ century revealed that alternative promoter usage is a widespread phenomenon, with a large fraction (maybe as much as 50%) of human protein-coding genes showing multiple alternative promoters [9]. The use of alternative promoters is compelling because of its impact on normal physiology and development. Unsurprisingly, the aberrant use of alternative promoters has been linked to many diseases, including cancer [10].

By exploiting CAGE and HelicoSCOPE sequencing technologies, Schmidl *et al*. profiled the transcriptome of different human T cell subsets from the conventional (T_CONV_) and regulatory compartments, and found a set of novel transcriptional start sites (TSS) that were present only in T_REGS_ [11]. Subsequent reporter assays and highly tailored PCR experiments suggested that FOXP3 has an alternative promoter, which is located 1.8kb upstream of its canonical promoter. Notably, a previous publication had already reported that the subject stretch of DNA showed promoter activity, exhibited directionality, and pointed in the same direction as FOXP3 [12]. Taken together, it is plausible that the transcriptional output of the human FOXP3 locus is richer than that of the current annotation, as, for example, a FOXP3 mRNA isoform could emerge from the T_REG_-exclusive promoter. Indeed, such transcripts are part of the FOXP3 gene model in the FANTOM CAGE-Associated Catalog (FANTOM-CAT), which is the most 5’-completed human transcriptome meta-assembly to date (S1 Fig). Furthermore, shorter transcripts that originate from the alternative promoter but do not extend further downstream until the last coding exon of FOXP3 are also part of the transcriptional model (S1 Fig). Therefore, the transcriptional output of the FOXP3 locus is still to this date not well understood.

Considering the biological implications associated with an alternative promoter and the lack of comprehensive characterization of the transcripts that emanate from the human FOXP3 locus, we set out to analyse its transcriptional output. By direct RNA sequencing (DRS) we identified a series of novel poly(A)+ transcripts emanating from both the canonical and the alternative promoters of FOXP3 in human T_REGS_. We investigated the coding capacity and subcellular localization of one of the unreported transcripts produced by the upstream promoter and it appeared to be non-coding, suggesting that FOXP3 could be classified as a bifunctional gene, an expression unit that generates both coding and non-coding products [13]. Interestingly, studies correlating FOXP3 RNA and protein expression at the single-cell level suggested that the upstream promoter may contribute to FOXP3 expression as part of a very intimate negative regulatory switch, adding yet another layer of complexity to the convoluted regulatory network operating at this locus. Interestingly, the highly cell type-specific activity of the promoter and its potential regulatory role may hold promise as biomarker and/or therapeutic target.

## 2. Results

### 2.1. Novel transcript isoforms originating from the human FOXP3 locus

The alternative promoter of FOXP3 is T_REG_–exclusive [11] and shows higher activity in naive T_REG_ cells after *in vitro* culture (S2A Fig). Thus, to survey the transcriptional output of the locus we performed DRS of cultured naive T_REG_ transcriptomes, and we used the classical combination of CD45RA and CCR7 as surface markers to sort this compartment. Because we observed that ~ 22% of circulating CD45RA^hi^CCR7^+^ T_REGS_ also expressed the activation/memory marker CD95 (S2C Fig), we selected the CD95^neg^ fraction (called *truly naive T*_*REG*_ or tnT_REG_) as target population to minimize any variability that could stem from differences in cell-type plasticity and expansion capacity (S2B Fig).

**Figure 1.**
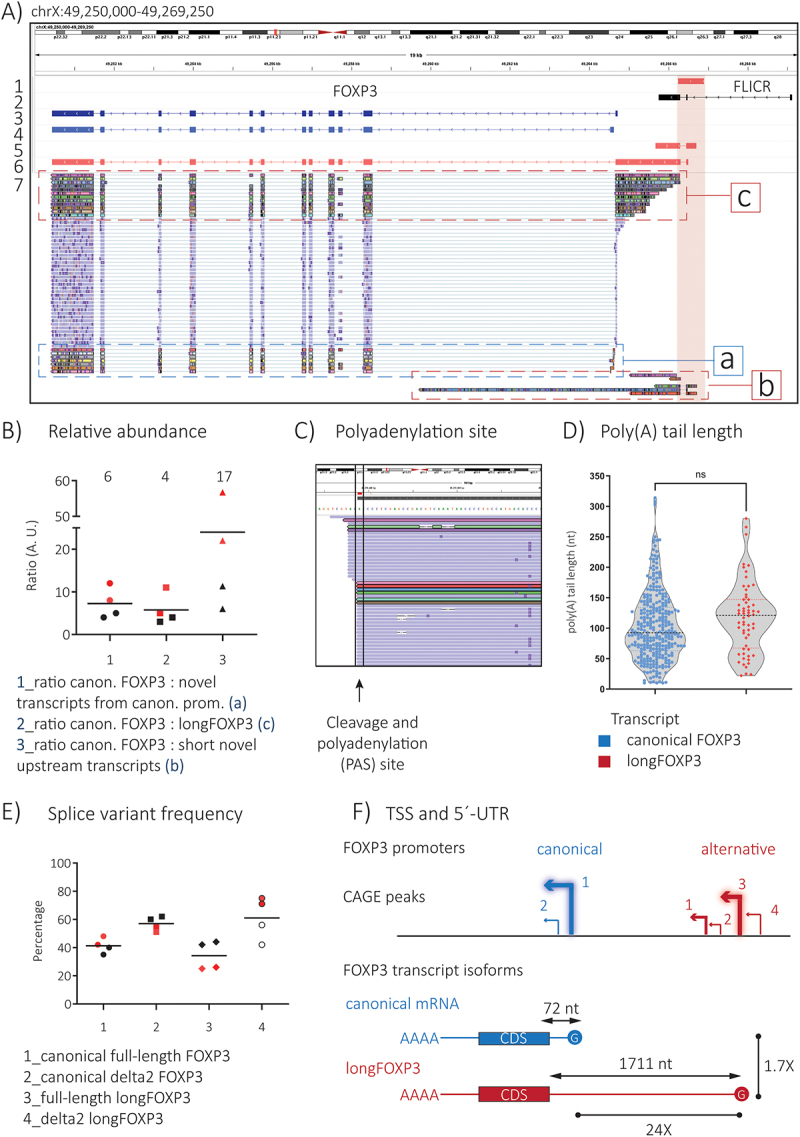
DRS revealed novel polyadenylated FOXP3-associated transcripts in expanded human T_REG_ transcriptomes. A) IGV browser screenshot of the human FOXP3 locus displaying nanopore DRS data. Alternative promoter of FOXP3, as described and used in reporter assays by Schmidl et al. (track 1), lncRNA FLICR isoform 3 (track 2), and full-length FOXP3 mRNA (track 3) are included as visual references for structure and genomic location. Track 4 shows a representative novel transcripts emanating from the canonical promoter. Tracks 5 and 6 show representative novel transcripts originating from the alternative promoter. Those that extended until the last annotated exon of FOXP3 were named longFOXP3 (track 6). Track 7 shows a selection of DRS reads. Due to limited space, only one out of four biological replicates and a small fraction of all relevant reads are shown. Please refer to S1 file for a merged BAM file containing all FOXP3 reads from the totality of the datasets. Canonical FOXP3 transcripts are colored light-blue, while novel transcripts are highlighted in bold. B) Approximate quantification of novel transcripts relative to canonical FOXP3 transcripts after selecting relevant reads. C) IGV screenshot zoomed into the annotated site of cleavage and polyadenylation of FOXP3 (red circle). A few relevant reads are displayed to exemplify the tight distribution around this genomic position. D) Distribution of the poly(A) tail length of canonical and longFOXP3 transcripts. Non-parametric Mann-whitney test. E) LongFOXP3 is mostly present in two splice variants: full-length and delta 2. F) Schematic representing the complexity of the alternative promoter. CAGE peak 3 was chosen as the representative TSS of longFOXP3 based on its reported CAGE score. (B) Values on top of each column are the median for the calculated ratios. (B) and (E), black dots correspond to 1-week-old T_REG_ cultures, while red dots correspond to over-4-week-old cultures.

The poly(A) fractions of total RNA collected from tnT_REG_ cultures were subjected to DRS. We detected several reads corresponding to the annotated full-length and delta 2 FOXP3 mRNA isoforms (Figure 1A). We expected to detect the lncRNA FLICR because it is both polyadenylated and expressed specifically in T_REGS_ [1]. However, we only observed a small number of reads that might represent 5´ decay products of FLICR (S5 Fig). Importantly, the DRS experiment revealed many novel transcript isoforms across the FOXP3 locus (Figure 1A). Here, the canonical promoter itself generated novel transcript isoforms with the same overall structure as the canonical FOXP3 mRNAs, except for the use of alternate structures for the first exon (exon −1) (‘rectangle a’ in Figure 1A and S3 Fig). Furthermore, the upstream promoter gave rise to transcripts that extended until the last coding exon of FOXP3 (‘rectangle c’ in Figure 1A). We named them *longFOXP3*. These were in agreement with the predicted transcripts found in the gene product model for FOXP3 in the FANTOM6 CAT assembly (S1 Fig). The upstream promoter also generated *shorter* transcripts (relative to longFOXP3) that terminated before reaching exon 11 of FOXP3, that is, their 3´-end mapped between the canonical and alternative promoters or within the first intron of the gene (‘rectangle b’ in Figure 1A).

We quantified the abundance of novel transcript isoforms relative to the most abundant product of the FOXP3 locus, i.e. the canonical FOXP3 mRNA (Figure 1B). Briefly, by comparing median values, the canonical transcript was six times (range: 4–12) more abundant than the novel transcripts originating from the same promoter, approximately four times more abundant than longFOXP3 (range: 3–11), and at least 17 times (range: 6–57) more frequent than the short upstream transcripts (Figure 1B).

### 2.2. LongFOXP3 is a potential FOXP3 mRNA isoform with a 5´-UTR extension

Because of its higher frequency compared to the other novel RNAs and the potential biological implications of such transcript, we focused our efforts on characterizing longFOXP3. We observed that longFOXP3 shares structural features with the canonical FOXP3 mRNA. Their 3´-ends showed a similar distribution of cleavage and polyadenylation sites centred at the annotated position (with minor variations of ±2 nt) (Figure 1C). Interestingly, poly(A) tail analysis revealed that FOXP3 reads as a whole had longer poly(A) tails (average modal poly(A) length: 89 As) than all transcripts analysed (average modal poly(A) length: 53 As) (S4C and S4D Fig), which may indicate greater stability [14]. LongFOXP3 reads showed a greater mean, modal, and median poly (A) tail length than the canonical FOXP3 transcript (S1 Table), but the difference was not statistically significant (Figure 1D). Finally, longFOXP3 appears to be subject to the same splicing processing as its canonical counterpart (Figure 1E). Two longFOXP3 splice variants were the most frequent, encompassing, on average, 93% of all longFOXP3 transcripts (range: 83% − 100%). One variant included all annotated coding exons of FOXP3 (named *full-length longFOXP3*, representing on average 37% of all longFOXP3 transcripts), while the other was missing exon 2 (named *delta2 longFOXP3*, which represented, on average, 56% of all longFOXP3 transcripts) (Figure 1E). Minor variants included transcripts in which exon 7 or exon 2 and exon 7 were spliced (not shown).

In contrast, longFOXP3 differed from the canonical transcript in terms of its 5´-end, showing a high variability in its starting position (Figure 1F). Such a 5´-end profile was expected as the upstream promoter was reported to be broad, consisting of a cluster of four defined TSSs (Table 1). Considering CAGE peak 3 as the main TSS because of its higher CAGE score (Table 1), longFOXP3 has a length of 3903nt and is ~ 1.7 times longer than its canonical counterpart.

**Table 1.** Robust FANTOM5 CAGE peaks associated to the FOXP3 locus.

Promoter	Peak number	CAGE cluster	TSS	Score
Canonical	peak 1	chrX:49264704–49264717	chrX:49264710–49264710	18671
	peak 2	chrX:49264549–49264582	chrX:49264567–49264567	315
Alternative	peak 1	chrX:49266299–49266300	chrX:49266300–49266300	396
	peak 2	chrX:49266450–49266454	chrX:49266452–49266452	195
	peak 3	chrX:49266491–49266500	chrX:49266496–49266496	453
	peak 4	chrX:49266722–49266750	chrX:49266729–49266729	261

The table includes the genomic coordinates and CAGE scores for the different TSS peaks identified by the FANTOM5 consortium. The CAGE peaks are located within the sequences used as promoters in reporter assays by Schmidl et al. [11].

### 2.3. LongFOXP3 does not code for a novel FOXP3 proteoform

**Figure 2.**
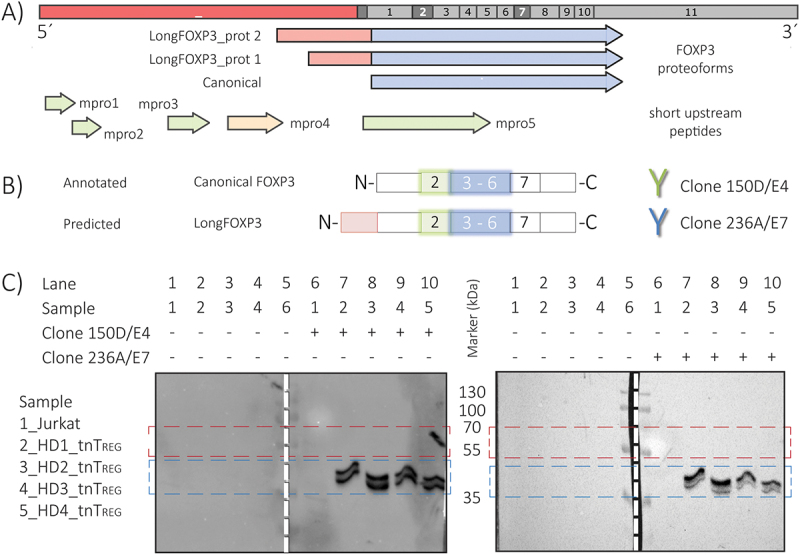
LongFOXP3 does not code for a novel FOXP3 proteoform. A) Scheme summarizing the coding potential of longFOXP3. Full-length longFOXP3 RNA is represented at the top. No 5´-cap nor 3´-poly(A) tail were included, annotated exons are indicated with numbers, and the 5´-UTR extension is represented by a red rectangle. The novel transcript could code for different FOXP3 proteoforms (long arrows): canonical transcription factor and predicted N-terminal extended proteoforms. Microproteins (mpro) that could be decoded from short upstream ORFs are also depicted and colour-coded according to their TIS (green arrow: CUG, orange arrow: AUG). B) Scheme representing both reported and predicted full-length FOXP3 proteoforms, and the antibodies used. Two monoclonal anti-human FOXP3 antibodies were used for WB. The antibody clone and the position of its epitope within the canonical FOXP3 protein (including the exons that code for that part of the protein) are colour-coded. C) Western blots of cultured T_REGS_. The existence of the predicted longFOXP3 proteoforms was tested in four biological replicates (HD1 – HD4). Jurkat cells were used as a biological negative control. To control for background signals, the samples were incubated with and without the primary antibody. The blots were first analysed using the antibody clone 236A/E7, and then stripped for analysis using the antibody clone 150D/E4. The blue rectangle on the blots highlights those bands interpreted as the canonical FOXP3 protein, whereas the red rectangle encircles the position in which the predicted longFOXP3 proteoforms are expected. The signal observed in lane 9 inside the red rectangle is attributed to the tweezers used to handle the blotted membrane.

Because longFOXP3 could constitute a FOXP3 mRNA isoform, we next sought to address its role as a coding transcript. Inspection of longFOXP3 revealed the potential to encode the canonical FOXP3 protein and two FOXP3 proteoforms with an N-terminal extension if considering CUG and AUG codons as possible alternative translation initiation site (TIS) [15] (Figure 2A). Because recent studies have reported the contribution of short ORFs to the translatome [16], we also searched for upstream ORFs (uORFs) and found several of these within the extended 5´-UTR (Figure 2A and Table 2 for a summary).

**Table 2.** Coding potential of longFOXP3.

Proteoform/microprotein	Variant	5`-UTR in which the start codon is located	START codon	Kozak score	Length (aa)	Calculated MW (kDa)
canonical FOXP3 protein	full length	Canonical	AUG	0.57	431	47.2
	delta2	Canonical	AUG	0.57	396	43.4
longFOXP3 proteoform_1	full length	Extended	AUG	0.49	539	60
	delta2	Extended	AUG	0.49	504	56.2
longFOXP3 proteoform_2	full length	Extended	CUG	0.56	593	66.4
	delta2	Extended	CUG	0.56	558	62.5
uORF1 → mprop1	NA	Extended	CUG	0.69	51	5.6
uORF2 → mprop2	NA	Extended	CUG	0.67	49	5.5
uORF3 → mprop3	NA	Extended	CUG	0.65	71	7.6
uORF4 → mprop4	NA	Extended	AUG	0.55	94	10.2
uORF5 → mprop5	NA	Canonical	CUG	0.4	218	22.5

The table summarizes all the peptides/microproteins and proteins that the longFOXP3 transcript could encode, and includes relevant features such as molecular weight (MW) and Kozak score. The Kozak score for each predicted translation initiation codon (TIC) provides an estimation of the likelihood of the ribosome engaging in translating the associated ORF.

To test the capacity of longFOXP3 to code for a novel FOXP3 proteoform, we analysed the FOXP3 proteoform profile of human T_REG_ cultures by Western Blotting (WB). Although the annotated full-length FOXP3 protein (FOXP3_fl) has a theoretical MW of 47.2 kDa, it is also detected in a range of 50–55 kDa in SDS-PAGE (see for example, antibody clone FJK-16s commercialized by ThermoFisher Scientific, catalogue number: sc -166,212). Therefore, to increase confidence, we investigated the band pattern using two commercial anti-human FOXP3 monoclonal antibodies (Figure 2B). The blots showed the characteristic band pattern linked to the detection of FOXP3 in human T cells: two bands closely spaced where observed when using clone 236A/E7, with the lower band (canonical FOXP3_∆2) being more intense than the upper band (canonical FOXP3_fl). The latter was more intense when using the full-length isoform-specific clone (blue rectangle in Figure 2C). Importantly, we did not observe any specific band within the expected size range for the predicted longFOXP3 proteoforms (red rectangle in Figure 2C and S6C and S6D Fig).

To further search for proof of the predicted microproteins and FOXP3 proteoforms, we reanalysed mass spectrometry data from Rieckmann *et al*., 2017 [17], which represents a comprehensive public proteomic dataset of human immune cells. After extensive analysis, we could not confidently identify the longFOXP3 proteoforms nor the uORF-encoded microproteins (S2 file).

### 2.4. LongFOXP3 does not code for any FOXP3 proteoform

**Figure 3.**
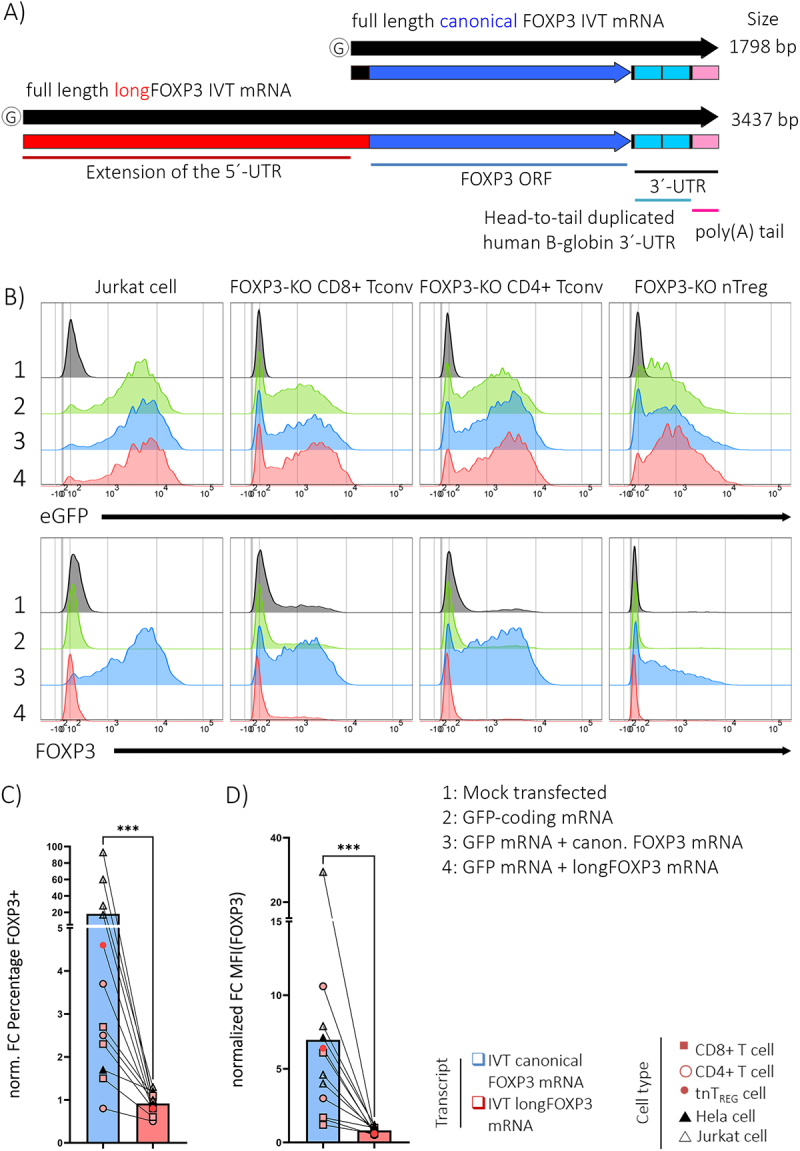
LongFOXP3 does not encode any FOXP3 proteoform. A) Schematic representation of the FOXP3 IVT constructs used for the overexpression assay. The thick black arrows represent the final product resulting from the IVT reaction. These are dissected into their constituting elements in the lines below. B) Results of an exemplary overexpression experiment to test the capacity of longFOXP3 to be decoded into a FOXP3 proteoform. In this case, Jurkat cells and different FOXP3^KO^ T cells were electroporated with IVT RNA emulating the full-length variant of the canonical or the longFOXP3 transcripts. The cells were co-transfected with a GFP-coding IVT mRNA for control of delivery and translation competence. Non-toxic equimolar amounts of the canonical and longFOXP3 transcripts were transfected (S7 Fig). C) Normalized Fold change in the percentage of FOXP3+ cells upon transfection with canonical FOXP3 or longFOXP3 constructs. D) Normalized Fold-change in the MFI of FOXP3 in the living singlet gate when cells were transfected with canonical FOXP3 or longFOXP3 constructs. Mock, mock-transfected. MFI, median fluorescence intensity. The MFI value corresponds to the entire living singlet gate and not to the FOXP3+ subpopulation. Non-parametric paired Wilcoxon test. *** *p* < 0.001.

We next applied a *gain-of-function* approach to test the capacity of longFOXP3 to code for a FOXP3 protein variant. To this end, cells were electroporated with *in vitro-*transcribed (IVT) RNAs corresponding to the full-length variant of the canonical FOXP3 mRNA or the full-length version of longFOXP3 (Figure 3A), and FOXP3 protein products were detected via flow cytometric analysis (Figure 3B).

We tested several different cell types in independent experiments. Namely, adherent and suspension human cell lines (HeLa, Jurkat and Raji cells), and primary human CD4+ or CD8+ conventional T cells. We detected FOXP3 signal above background levels when we transfected the canonical FOXP3 mRNA but not longFOXP3 (Figure 3C,D). T_REGS_ have been reported to deploy a non-canonical DAP5/eIF3d-dependent mechanism to achieve essential mRNA translation [18]. Therefore, we used FOXP3-knockout T_REG_ (FOXP3^KO^ T_REG_) cultures generated using CRISPR technology (S8 Fig) to reduce the background signal originating from the genome-encoded protein. Jurkat cells and conventional CD4+/CD8+ T cells with FOXP3 gene deletion were included as controls. Figure 3B shows the analysis of the internal positive control (GFP) and the protein of interest (FOXP3) in the last experiment. In summary, while FOXP3 protein signal was readily detected above background level when transfecting the canonical FOXP3 mRNA, no additional FOXP3 protein could be detected with the longFOXP3 IVT transcript (Figure 3C,D).

### 2.5. The activity of the alternative promoter is heterogeneous in T_REG_ cultures and shows an inverse relationship with FOXP3 protein levels

As our available evidence indicated that longFOXP3 was non-coding, we considered the possible regulatory role of the alternative promoter. To test the possibility of transcriptional interference (TI) [19], we visualized the levels of transcripts from each promoter and the total amount of FOXP3 protein simultaneously at the single-cell level. via the PrimeFlow assay. See S10 Fig. for a detailed description of the probes used.

**Figure 4.**
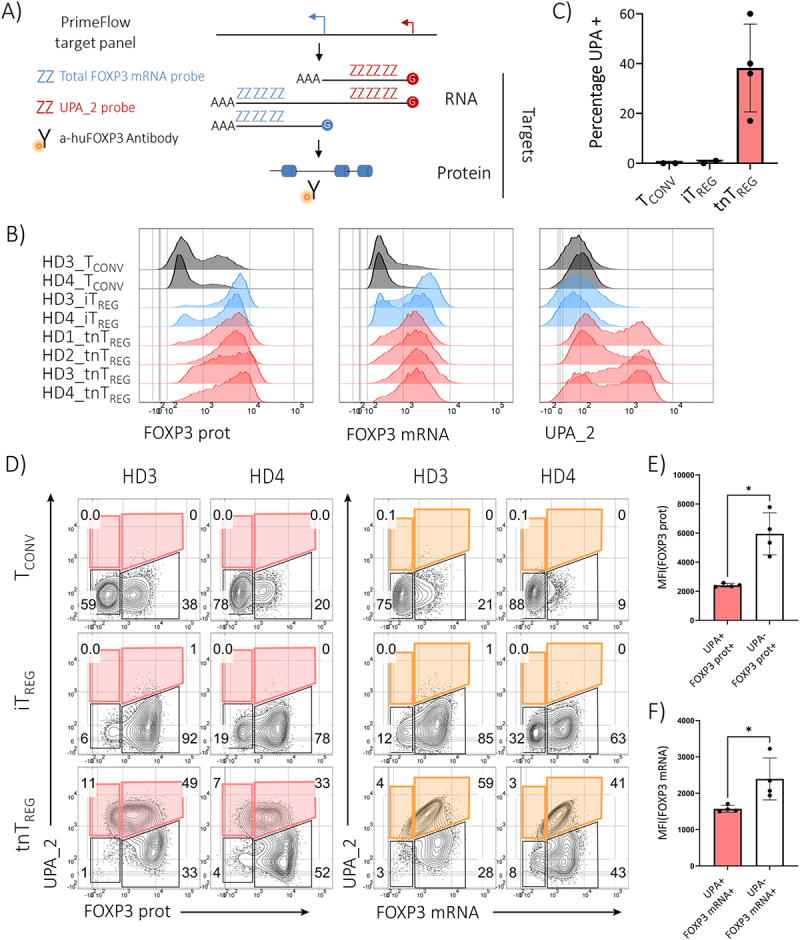
The alternative promoter of FOXP3 appears T_REG_-exclusive, but it is not active in every cell in culture. A) Scheme explaining the amplification strategy used by both the PrimeFlow and RNAscope assays and summarizing the RNA species expected to be detected using both PrimeFlow probes, ordered from left to right in terms of decreasing abundance, as observed in the DRS experiment (Figure 1). The panel design allowed the co-detection of total FOXP3 protein, total FOXP3 mRNA, and transcriptional activity of the alternative promoter. B) Expanded tnT_REG_ cells were reactivated under normal culture conditions. In parallel, expanded naive-like T_CONV_ cells were reactivated either in the presence of rhIL-2 or rhIL-2 and rhTGF-b1 (iT_REG_ condition). FACS plot are pre-gated on living RPL13a+ singlets and different marker combinations according to the sample. A total of four tnT_REG_ cultures were analyzed; two are shown here. Histograms of all relevant targets analyzed across the three (CD4+) T cell cultures. Black histograms: reactivated T_CONV_; light blue histograms: iT_REG_ cells; red histograms: tnT_REG_ cultures. C) Percentage of UPA+ cells across T cell cultures. D) Contour plots showing how the activity of the upstream promoter correlates with the other targets (FOXP3 protein and total FOXP3 mRNA). E) MFI of FOXP3 protein or F) MFI of FOXP3 mRNA in cells that either showed transcriptional activity from the upstream promoter or did not. Only the values for the tnT_REG_ cultures are plotted. E-F) Nonparametric unpaired Mann-Whitney test. * *p*<0.05.

We detected FOXP3 mRNA and protein in reactivated conventional T cells with an increase in the percentage and MFI of both parameters in the iT_REG_ cultures, but UPA_2 signal was only present in bonafide T_REGS_ (tnT_REGS_ in Figure 4B). Notably, the activity of the upstream promoter was not homogeneous across the culture. On average, approximately 40% (range: 20%—60%) of cells showed UPA_2 signal (Figure 4C), and UPA_2^pos^ cells had differences in signal intensity of over one order of magnitude. The activity of the upstream promoter seemed to show an inverse relationship with total FOXP3 protein, suggested by the lower MFI of FOXP3 protein observed in cells that were positive for the *UPA_2* probe (Figure 4D,E). In accordance, the *UPA_2* signal also showed an inverse relationship with the levels of total FOXP3 mRNA (Figure 4D,F).

### 2.6. The alternative promoter is more active in cultured naive T_REGS_ compared to memory T_REGS_

**Figure 5.**
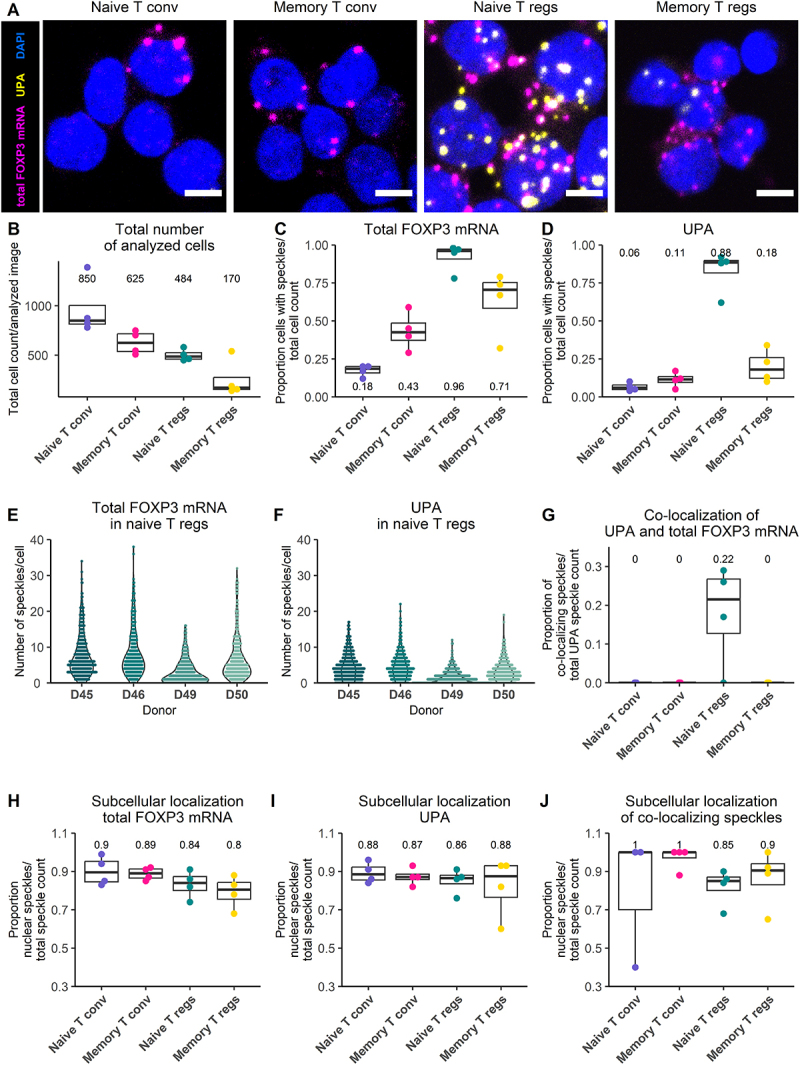
The alternative promoter is more active in naive T_REG_ cultures than in their memory counterpart, and short and long transcripts can be found in the nucleus and the cytoplasm. Four T cell cultures (naive (−like) T_CONV_, memory T_CONV_, (truly-) naive T_REG_, and memory T_REG_) were analyzed with the RNAScope assay using the total FOXP3 mRNA and UPA_Scope probes described in S10 Fig. A) Confocal microscopy overlay of total FOXP3 mRNA (magenta), UPA_Scope (yellow), and DAPI (blue) in representative zoomed-in regions showing T cells from the four different cultures analysed (from left to right, scale represents 5 µm). B) Total number of cells analysed per T cell culture. C) Proportion of cells that showed total FOXP3 mRNA speckles in each T cell culture. D) Proportion of cells that showed UPA_Scope speckles in each culture condition. E) Number of total FOXP3 mRNA speckles per cell in naive T_REG_ cultures from different biological replicates. F) Number of UPA_Scope speckles per cell in naive T_REG_ cultures from different biological replicates. G) Proportion of UPA_Scope speckles that co-localize with total FOXP3 mRNA speckles in each cell type. These events were interpreted as longFOXP3 transcripts. H) Proportion of all total FOXP3 mRNA speckles with intra-nuclear localization. I) Proportion of all UPA_Scope speckles with intranuclear localization. J) Proportion of all co-localization events with intranuclear localization. B) – J) Values at the top or bottom of each column represent the median of the group for the plotted variable of the respective condition.

Based on the number of DRS reads that could be ascribed to each promoter (Materials and Methods), the output of the canonical promoter was approximately five times greater than that of the alternative promoter. Therefore, the high prevalence of activity of the upstream promoter within T_REG_ cultures (Figure 4C), and the high signal intensity observed was very surprising. Although we did not capture FLICR in our DRS experiment, this discrepancy could have stemmed from co-detection of this lncRNA (See S10 Fig). Therefore, we characterized the output of the upstream promoter at the single-cell level using the RNAscope assay with the same total FOXP3 mRNA probe, but we re-designed the UPA probe to avoid detection of FLICR (see UPA_Scope probe in S10 Fig). To emulate the expression profiling performed by Schmidl *et al*., we studied the activity of the alternative promoter in cultures of truly naive and memory T_REGS_, as well as in naive-like and memory CD4+ conventional T cells (Figure 5).

The RNAscope assay reaches single-molecule detection level which is observed as fluorescent *foci* or speckles (Figure 5A). Segmentation of total FOXP3 mRNA speckles and UPA_2 speckles and quantification of the spot count per cell revealed that the percentage of cells showing at least one total FOXP3 mRNA speckle was higher in the T_REG_ cultures than in the T_CONV_ cultures (Figure 5C). Importantly, in agreement with Schmidl *et al*., naive T_REGS_ showed a higher percentage of cells with UPA_Scope speckles compared to T_CONV_ cells and to their memory counterpart. We observed a median of 88% UPA_Scope^pos^ cells in naive T_REG_ cultures that sharply contrasted with a median of only 6%, 11%, and 18% UPA_Scope^pos^ cells in naive T_CONVS_, memory T_CONVS_ and memory T_REGS_, respectively (Figure 5D and S2A Fig). Comparison of the proportion of cells that contained speckles or not between any two T cell types (conditions) by means of a logistic regression-based statistical test for contingency tables (see Materials and Methods) showed that all conditions were statistically significantly different (S4–5 Table).

To correlate RNA and protein levels an aliquot of the cultured cells was analysed by FACS for total FOXP3 protein (S12B-C Fig). Interestingly, memory T_REGS_, which showed a smaller percentage of cells with *UPA_Scope* speckles (Figure 5D), exhibited a clear trend of higher MFI for FOXP3 protein compared to naive T_REG_ cultures (S12C Fig).

### 2.7. Rnascope reveals that the alternative promoter mostly outputs short transcripts

Next, to profile the output of the alternative promoter, we performed co-localization analysis, where co-localizing signal of juxtaposed total FOXP3 mRNA and UPA_2 speckles was interpreted as the longFOXP3 transcript. In correspondence with a high percentage of cells with UPA_Scope speckles and a wide distribution in the number of total FOXP3 mRNA and UPA speckles per cell (Figure 5E,F), naive T_REGS_ showed the highest percentage of co-localization events across all T cell cultures (Figure 5G). Unexpectedly, the median percentage of UPA speckles that co-localized with the *total FOXP3 mRNA* probe was only 22 (range: 0% −30%) (Figure 5G). The abundance of total UPA_Scope-only events relative to longFOXP3 (co-localization events) was, on average, approximately three (range: 1.8–5.3) (Table 3).

**Table 3.** Abundance of short upstream transcripts relative to longFOXP3.

Donor	Total count co-localization events (longFOXP3)	Total count UPA_Scope-only events	Ratio UPA_Scope-only : co-localization
HD45	850	1713	2.0
HD46	737	1347	1.8
HD49	122	647	5.3
HD50	391	1151	2.9

Total numbers of UPA_Scope-only and co-localization events for each donor were used to profile the output of the alternative promoter in terms of short upstream transcripts and longFOXP3 RNAs.

### 2.8. Short and long upstream transcripts are found in the nucleus and the cytoplasm

The large majority of all speckles (magenta, yellow, and white) were found within the nucleus (Figure 5H–J). Taking into account that underestimation of cytoplasmic transcripts was inherent to our analysis and focusing on naive T_REG_ cultures because they showed a much higher number of relevant events, we could report that at least 15% of longFOXP3 (range 10% −30%) and 15% of UPA speckles (range: 10%–25%) are exported to the cytoplasm.

### 2.9. Potential non-adenylated FOXP3 transcript isoforms in human T_REGS_

The majority of the RNA species emanating from the upstream promoter were not co-detected by the *total FOXP3 mRNA* probe (Figure 5G and Table 3), suggesting that the alternative promoter mostly outputs short RNAs instead of longFOXP3 transcripts. However, the higher prevalence of short upstream transcripts over longFOXP3 observed with the RNAscope assay, which targeted all RNA species, was clearly at odds with the results of the DRS experiment, which reported mostly poly(A)+ transcripts (S4B Fig). Therefore, considering that not all mRNAs, and certainly not all lncRNAs, are polyadenylated [20], we reasoned that the main output of the upstream promoter could be *short* non-adenylated transcripts.

**Figure 6.**
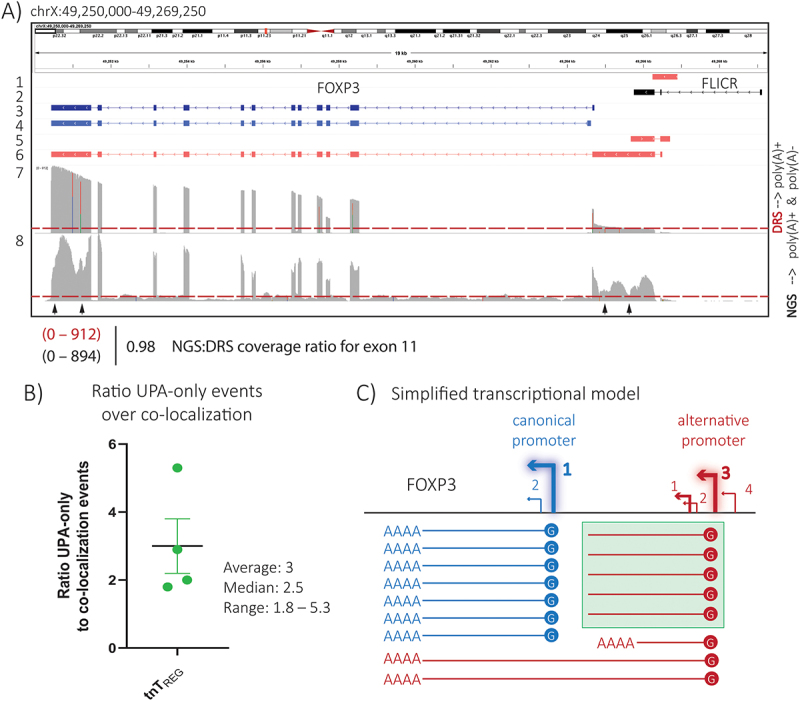
Potential non-adenylated FOXP3 transcript isoforms in expanded human T_REGS_. A) IGV browser screenshot displaying sequencing coverage of the human FOXP3 locus using 2nd and 3rd generation sequencing technologies applied to the same RNA sample from human tnT_REG_ cultures. For genomic location and structural reference: track 1, alternative promoter as described and used in reporter assays by Schmidl et al.; track 2: lncRNA FLICR isoform 3; track 3, canonical full-length FOXP3 mRNA isoform; track 4, representative novel transcript from canonical promoter (S3 Fig); track 5, representative novel short upstream transcript; and track 6, full-length longFOXP3. Track 7 displays the coverage of the DRS experiment described in Figure 1 (pre-processing: poly(A)+ enrichment), while track 8 shows the coverage of the short-read RNA-seq experiment (pre-processing: ribo-depletion). The heights of the coverage tracks are the same. The red dotted line indicates the approximate maximum coverage for the region of possible transcription initiation for CAGE peak 1 observed with DRS. The black arrows indicate regions in which the coverage was lower in the NGS experiment. B) Ratio of longFOXP3 transcripts (co-localization events) to short upstream transcripts (UPA-only events) as observed in the RNAscope analysis (Figure 5G and Table 3). C) Plausible transcriptional model of the human FOXP3 locus. The upstream promoter outputs two classes of transcripts (represented by transcripts with a red 5´-cap): (i) non-adenylated RNAs that do not extend sufficiently into the FOXP3 gene to be simultaneously detected with the totalFOXP3 mRNA probe in addition to the UPA_Scope probe (green box), and (ii) polyadenylated RNAs that mostly extend until exon 11 of FOXP3 (longFOXP3), using the same PAS site as those FOXP3 transcripts originating from the canonical promoter (blue 5´-cap). Short poly(A)+ transcripts are also generated by the upstream promoter, but these are rare. Upstream non-poly(A) transcripts are more abundant than poly(A)+ transcripts generated from the same promoter, but are readily missed by the standard DRS protocol. For simplicity, the lncRNA FLICR is not included, novel transcripts originating from the canonical promoter are not discriminated, and the relative abundances between the different RNAs are only approximate.

To test this hypothesis, we performed classical Illumina RNA-seq on an rRNA-depleted RNA sample prepared from a standard T_REG_ culture. To minimize the impact of biological variation and make the DRS and NGS results as comparable as possible, we used an aliquot of the same RNA sample subjected to the long-read experiment described in Figure 1. Figure 6A compares the coverage of the FOXP3 locus when DRS or NGS was applied to the same RNA sample generated from cultured T_REG_ cells. Both sequencing experiments displayed a higher coverage towards the end of the FOXP3 gene (3´-end enrichment). The RNA-Seq dataset showed the expected higher intron coverage associated with the capture of nascent (i.e. incompletely spliced) RNAs and a series of drops in coverage that we suspect were due to some sequence factor (e.g. GC content) that specifically affected Illumina-seq.

The coverage along exon 11 of FOXP3 was almost the same using both sequencing technologies (coverage NGS_exon 11_: 0–894, coverage DRS_exon 11_: 0–912, ratio coverage NGS:DRS = 0.98), allowing direct visual comparison of the datasets without the need for normalization. Notably, we observed an increase in coverage across the stretch between the canonical and the upstream promoter in the RNA-Seq dataset compared to DRS with no strand bias upon visual inspection of the individual RNA-Seq reads. This increase in coverage supports the hypothesis that the alternative promoter outputs a mixture of adenylated and short non-adenylated transcripts. We suspect that the putative non-adenylated transcripts end somewhere close to the canonical promoter region, as we observed no obvious increase in coverage within the first intron of FOXP3 that could correspond to predicted transcripts like ENCT00000477108.C1 (track 8 in S1 Fig).

Based on our RNAscope analysis, we expected the short non-adenylated upstream transcripts to be approximately two to five times more frequent than the adenylated RNAs generated by the upstream promoter (represented mostly by longFOXP3) (Table 3 and Figure 6B). Accordingly, combining the DRS and RNA-seq results, we calculated that the ratio of non-adenylated to adenylated transcripts originating from the upstream promoter could be up to four (see Materials and Methods). The schematic in Figure 6C puts forward a simplified model of the transcriptional output of the human FOXP3 locus, considering only the canonical and the alternative promoters.

## 3. Discussion

### 3.1. Summary of results

Our data revealed that the transcriptional output of the human FOXP3 locus is far more complex than that currently described in the main public databases *NCBI Gene* and *EMBL/EBI Ensembl*. Using DRS, we identified novel adenylated transcripts emanating from both the canonical and the alternative promoters of FOXP3 in cultured human T_REGS_. The novel upstream transcripts included *short* and long RNA strands, with the latter sharing many structural features with the annotated mRNA, except that they harboured an extended 5´-UTR (longFOXP3). Further analysis using classical RNA-Seq suggested that the alternative promoter also generates short non-adenylated transcripts that are more abundant than the poly(A)+ species emanating from the same promoter. Accordingly, profiling of the alternative promoter with RNAscope suggested that it preferentially outputs *short* transcripts. Considering the distribution of total FOXP3 mRNA and UPA speckles per cell and that we analysed approximately 500 naive T_REG_ cells in four biological replicates, the rather scarce co-localization was unlikely to stem from a sampling problem.

*In silico* analysis of longFOXP3 revealed a broad coding potential. LongFOXP3 could encode the canonical FOXP3 protein, FOXP3 proteoforms with an N-terminal extension, and/or different microproteins. RNAscope analysis showed that at least 18% of longFOXP3 is exported to the cytoplasm, which is in line with a coding function. However, several analysis with orthogonal techniques argued against its role as a messenger transcript. Translational inhibitory elements present in the extended 5´-UTR could explain the lack of decoding into at least one FOXP3 proteoform. For example, multiple ORFs located upstream of the coding sequence can impinge on protein expression [21], and G-quadruplexes in the extended 5´-UTR could mark the transcript for decay in P-bodies [22]. In addition to uORFs, we predicted two G-quadruplexes in the 5´-UTR of longFOXP3 (but none in the sequence shared with the canonical mRNA) using the algorithm G4Hunter (data not shown).

PrimeFlow and RNAscope assays suggested that the alternative promoter is only functional in T_REG_ cells and revealed that the alternative promoter is more active in expanded truly naive T_REGS_ compared to their memory counterpart. Interestingly, the PrimeFlow assay suggested an apparent negative relationship between the activity of this promoter and total FOXP3 mRNA and protein levels. A similar conclusion was drawn from the RNAscope assay, although the separate detection of protein and RNA prevented the establishment of a proper correlation. Nevertheless, both experiments would point to TI as a plausible regulatory mechanism of the human FOXP3 locus, a phenomenon that has been previously reported for other human genes like the immune ligand MICA [23].

### 3.2. Open questions

Our study clearly demonstrated that the human FOXP3 locus produces many more transcript isoforms than currently annotated and lays the foundation for future endeavours to address key research questions. It is critical to identify a phenotype associated with activation of the upstream promoter as it would be the first indication that the alternative promoter is a key regulator of the FOXP3 locus and not simply a transcriptional eccentricity. Furthermore, it would enable functional assays.

Further studies are needed to confirm the occurrence of TI, which would entail manipulating the endogenous FOXP3 locus. Considering that 5–20% of CRISPR-Cas9-edited T cells experience partial or complete loss of the targeted chromosome and persist for weeks in the cultures [24], switching off the alternative promoter using CRISPR interference (CRISPRi) technology [25] or preventing readthrough of the canonical promoter using antisense oligonucleotides (ASOs) or LNA GAPmers [26] to promote early transcriptional termination would be preferable. If TI regulates the FOXP3 locus, it is critical to identify the precise mechanism of interference and its induction. In this regard, multiple TFs are predicted to bind to the upstream promoter, which could either promote or prevent TI. If we assume that the FOXP3 locus is subjected to TI, this creates a conundrum: how do the multiple enhancers that promote FOXP3 expression ‘decide’ to act on the right promoter, considering that the result would be diametrically opposite? Furthermore, why is the alternative promoter more active in expanded naive T_REGS_ than in cultured memory T_REGS_

Finally, the putative non-adenylated transcripts generated from the upstream promoter require further investigation. Until this point, their sequence can only be inferred from the coverage pattern observed in our short-read RNA-Seq data. We envision an approach that includes rRNA-depletion plus poly(A) depletion of a T_REG_–derived RNA sample, followed by column-based size selection and *in vitro* poly(A)-tailing before DRS library preparation to address their existence and exact structure. If their existence was confirmed, their function could be tested by overexpression (from either the endogenous locus or a vector) in a FOXP3^KO^ T_REG_ background in combination with transcriptomic profiling.

The regulation of FOXP3 is complex, with mechanisms working at every step of the expression pathway. The results presented in this work suggest that even after almost a quarter of a century of profuse study of this gene since it was first cloned in 2000 [27], there might still be much to uncover. The inverse relationship between the activity of the upstream promoter and the total amount of mRNA and total FOXP3 protein combined with the poor translatability of the IVT version of longFOXP3 points to an intimate negative self-regulatory loop. It is reasonable to hypothesize that, together with other mechanisms, the alternative promoter helps T_REG_ cells fine-tune FOXP3 levels and hence modulate the functionality of the cell. The inclusion of translational repression of longFOXP3 would point to FOXP3 as the second gene subject to LUTI regulation in humans after the oncogenic MDM2 gene [28]. Intriguingly, as the alternative promoter is only active in T_REGS_, this mechanism would only operate in this compartment, and we observed it under the standard culture conditions used for this cell type.

From an applied science point of view, the T_REG_-exclusive activity of the alternative promoter could be harnessed as a biomarker for this cell type. Furthermore, the apparent negative effect of this promoter on the output of the canonical promoter, and presumably on the suppressor fitness of the cell, could be exploited in a clinical setting to boost or hinder T_REG_-mediated suppression, for example, in organ transplant or cancer patients, respectively. Development in antisense oligonucleotide technology and RNA-targeting small molecules have proven that ncRNA is a ‘druggable’ molecule amenable for therapeutic treatment [29] and FOXP3 RNA has recently been reported to be a viable ASO target in a mouse cancer model [30].

### 3.3. Limitations of the study

One limitation of the study was inherent to the DRS protocol used, which does not allow discrimination of capped transcripts from decay products or abortive reads and prevents proper annotation of the novel transcripts. Furthermore, it only provided a rough estimate of the relative output of both promoters. This can be addressed by resorting to TERA-seq [31] or Nanopore ReCappable sequencing (NRCeq) [32,33], which are recent adaptations of the DRS protocol that enable *actual* full-length sequencing.

Overexpression assays suggested that longFOXP3 was not decoded into any FOXP3 protein. However, the IVT constructs might have not perfectly resembled the endogenous transcripts as only one out of four possible TSS was chosen without knowing which the main one was, and any epitranscriptomic remodelling that could take place on the endogenous transcript was not incorporated, i.e. the longFOXP3 IVT RNA lacked ‘nuclear’ experience that might have been necessary to render it translatable [34].

Finally, the microscopy-based quantification of the transcripts emanating from the FOXP3 locus by counting spots suffered from limited accuracy due to the consideration of obvious agglomerates as one transcript, the overestimation of nuclear localization associated with the 2D projection of a 3D system, which is expected to be higher in lymphocytes (i.e. cells that tend to have a relatively high nucleus-to-cytoplasmic (N:C) ratio), and the inability to discriminate nascent longFOXP3 transcripts from short transcripts and decay products from intact transcripts. Therefore, the results regarding abundance, subcellular distribution, and percentage of co-localization should be considered only semi-quantitatively.

## 4. Materials and methods

### 4.1. Cell lines, primary T cell isolation and culture

Human Peripheral Blood-derived Mononuclear Cells (PBMCs) were isolated from healthy donors by standard density gradient centrifugation employing Ficoll-Paque PLUS. The origin of the blood was either fresh blood or leukapheresis products purchased from the blood bank, Institut für Transfusionsmedizin (ITM) Charité- Universitätsmedizin Berlin.

Before FACS sorting, PBMCs were subject to positive enrichment using different magnetic bead-based kits (pan T cells or CD4+ cells, Miltenyi Biotec). Column-based enrichment was performed on an autoMACS Pro Separator using the *PosselS* program for maximum recovery. All FACS sorting was performed on a FACSAria II cell sorter (BD Biosciences).

For culture experiments, cells were incubated at 37°C in 5% CO_2_. Jurkat cells were normally cultured in T25 or T75 culture flasks with standard RPMI-1640 medium, supplemented with GlutaMAX (2 mm), FCS (10% v/v), Penicillin/Streptomycin (1% v/v) and Beta Mercaptoethanol (50 µM) at a concentration of 0.5–3 X 10 [6] cells/mL. T_CONV_ cells (CD4+ or CD8+) were plated at a density of 1 × 10[6] cells/mL in standard T_CONV_ medium (RPMI-1640 supplemented with GlutaMAX 2 mm, FCS 10% v/v, Penicillin/streptomycin 1% v/v, and 2-mercaptoethanol 50 µM) in 96-multiwell round-bottom culture plates and initially activated using the human T cell expansion kit (Miltenyi Biotech, 130-091-441) following the manufacturer recommendations. When appropriate, expanded CD4+ T_CONV_ were further cultured in medium supplemented with rhIL-2 (100 IU/mL) or rhIL-2 and rhTGF-β1 (5 ng/mL) (iT_REG_-promoting conditions) and restimulated using T cell TransAct (Miltenyi Biotech, 130-111-160) following the manufacturer recommendations. T_REG_ cells were plated at a density of 5 × 10[5] cells/mL in T_REG_ medium (TexMACS -Miltenyi Biotech, 130-097-196-, human AB serum 5%, rhIL-2 100 IU/mL, Rapamycin 100 nM, and Penicillin/Streptomycin 1%) in 96-multiwell round-bottom culture plates and initially activated using human T_REG_ expansion kit (Miltenyi Biotech, 130-095-353) following the manufacturer recommendations. When appropriate, expanded T_REG_ cells were restimulated with T_REG_ expansion kit or T cell TranscAct.

### 4.2. Sequencing

#### 4.2.1. RNA extraction

The TRIzol approach was preferred because of its better RNA yield with no bias in terms of molecular size nor base composition. The protocol described in TRIzol Reagent User Guide Doc. Part No. 15596026.PPS, Pub. No. MAN0001271, Rev. C.0 was followed. No DNase treatment was conducted. Samples were stored at −80°C until use. RNA samples were thoroughly QCed: protein and salt contamination was measured using UV/Vis-spectroscopy (NanoDrop 1000 Spectrophotometer), while integrity was assessed on Agilent`s TAPE station system right before processing for library preparation.

#### 4.2.2. Direct RNA sequencing (DRS)

Total RNA samples were enriched for poly(A)+ transcripts using Dynabeads mRNA Purification Kit (Invitrogen, catalogue number 61,006) following the manufacturer`s instructions. Between 75 and 100ng of poly(A)+ RNA were used for library preparation using the SQK-RNA002 or the SQK-RNA004 kit following the manufacturer`s instructions. Sequencing of RNA002 libraries was performed using FLO-MIN106 (R 9.4.1) flow cells run on a GridION device (Oxford Nanopore Technologies) while RNA004 libraries were sequenced using FLO-MIN004RA flow cells on a MinION mk1b. The sequencing was stopped once the ‘read count’ curve plateaued (real-time check of the sequencing performance). This time point was typically 24 hours after start. Following high-accuracy basecalling with either Guppy (5.0.11, and MinKnow 21.05.8) for RNA002 or Dorado v0.6.0 (rna004_130bps_hac@v3.0.1 model) for RNA004, DRS reads were aligned to human genome hg38 (GRCh38 Genome Reference Consortium Human Build 38, Homo sapiens) using minimap2 [35] with the -axe splice parameter. The resulting SAM files were parsed to sorted, indexed BAM files using SAMtools (v1.7, [36]) with only primary alignments (flag 0 & 16) retained. Visualization of alignments across the FOXP3 locus was performed using the Integrative Genomics Viewer (IGV, [37]). Poly(A) tail estimates for all reads in each RNA004 dataset were obtained from Dorado v0.6.0 while the BBmap [38] *filterbyname.sh* module was used to estimate poly(A) tail lengths for individual FOXP3 isoforms. Visualization of poly(A) tail length distributions was performed using the statistical software R v4.3.2 [39] with RStudio as graphical interface [40]. The data was read in using functions of the ‘data.table’ package [41] to create a data frame and processed using the ‘dplyr’ package [42] for input into the graphical plotting package ‘ggplot2’ [43]. Following this the graphs were arranged for visualization using the ‘patchwork’ package [44].

#### 4.2.3. Illumina short-read sequencing

RNA-Seq libraries were prepared using the NEBNext Ultra II Directional RNA Library Prep Kit for Illumina in conjunction with the NEBNext rRNA Depletion Kit v2 (Human/Mouse/Rat). 760 ng of total RNA was used as input and the resulting libraries were sequenced using an 600 cycle kit (v3 chemistry) in paired-end mode on an Illumina MiSeq. Resulting basecalled FASTQ files were subject to adaptor and quality trimming using TrimGalore v0.6.5 (https://github.com/FelixKrueger/TrimGalore) with the quality cut-off parameter set to 30. Trimmed reads were aligned to the HG38 genome using STAR aligner (v2.7.9, [45]) with the resulting SAM file parsed to a sorted, indexed BAM with only read pairs in a proper alignment retained. Visualization was again performed using IGV.

#### 4.2.4. Analysis of sequencing data

To determine the abundance of novel transcripts relative to canonical FOXP3 mRNA we used a set of criteria to filter out DRS reads (S12A Fig). Namely, (i) reads whose 5´-end mapped to the promoter region of PPP1R3F, (ii) Reads whose 5´-end mapped outside the regions of probable transcription start (defined below in S5B Fig) were interpreted as decay products or the outcome of aborted sequencing, (iii) Reads with 3´-UTRs that were 50 nt shorter than the annotated one. This means that their 3´-end most nucleotide mapped to a position upstream of *chrX:49,250,487–49,250,487* (iv) Reads in which the FOXP3 ORF was incomplete or incorrect due to aberrant splicing or reads corresponding to immature transcripts.

While the canonical promoter is a cluster of two TSS in which one is clearly more prominent than the other (CAGE score ratio peak1 : peak2 = 59X), the alternative promoter is a cluster of four closely located TSSs with none of them showing an overt predominance (Table 1). Because the DRS protocol used cannot sequence the last 5–15 nucleotides of the 5´-end [31], and mRNA mostly decays in a 5´ → 3´ fashion [31], we could not reliably ascribe one transcript to a particular TSS within the 4-peak cluster and thus define the one most frequently used. To circumvent this limitation, we resorted to public CAGE data. Owing to its CAGE score, we chose peak 3, although it was only ~ 12.5% higher than the second peak with the highest score, peak 1 (Table 1).

To assign FOXP3 reads to a particular promoter we defined *regions of probable transcription initiation* by using public CAGE data (*source: FANTOM5 CAGE peaks, robust set track in NCBI´s genome data viewer https://www.ncbi.nlm.nih.gov/gdv/browser/genome/?id=GCF_000001405.40*)) and applying a series of assumptions (S12C Fig). The steps were as follows: (1) the reported CAGE peaks were broadened by five times their standard deviation. The value was decided based on the maximum upstream dispersion of the reads that could be assigned to the minor CAGE peak within the canonical promoter of FOXP3. Asymmetry of the CAGE peak was considered. (2) A final correction is applied by subtracting 10 nucleotides because of the inherent limitation of the DRS technology (i.e. the broadened CAGE peaks are shifted 10nt downstream). The average value of the reported range of uncertainty was chosen due to what it was observed for the reads that could be assigned to the main TSS within the canonical promoter.

To calculate the output of the alternative promoter relative to the output of the canonical promoter based on DRS data only, we counted all reads that could be ascribed to the latter (i.e. canonical FOXP3 mRNA and novel transcripts discussed in S3 Fig) and divided this number by the total amount of reads that we could confidently assign to the alternative promoter. Expressed as an equation, this is *ratio output canonical promoter : output alternativepromoter = (reads*_*canonical FOXP3 mRNA*_ *+ reads*_*novel transcript from canonical prom.*_*)/(reads*_*longFOXP3*_ *+ reads*_*short upstream transcripts*_).

To profile the output of the alternative promoter we calculated the ratio of non-adenylated to adenylated transcript by combining the DRS and NGS datasets together with a series of assumptions. Namely,

- the alternative promoter outputs two kinds of transcripts: adenylated and non-adenylated. *Alt. prom. output = Alt. prom. poly(A)*^*pos*^ *output + Alt. prom. poly(A)*^*neg*^ *output*
- In the DRS dataset the output of the alternative promoter includes adenylated short RNA strands (rectangle b in Figure 1A), (intact or degraded) longFOXP3, and special splicing variants of longFOXP3. Short RNAs and special splicing variants of longFOXP3 are infrequent and their contribution to the DRS dataset is negligible. *Alt. prom. poly(A)*^*pos*^ *output* (133 reads) *= longFOXP3* (126 reads) *+ special splicing variant longFOXP3* (4 reads)*+ short upstream transcripts* (3 reads) *≈ longFOXP3*.
- In both sequencing datasets (DRS and NGS), the coverage of the canonical promoter region is the result of sequencing intact canonical FOXP3 mRNA and longFOXP3 transcripts (either intact or degraded). *coverage over canon. prom. = output of canonical transcripts* (206 reads) + *intact longFOXP3* (39 reads) *+ degraded longFOXP3* (87 reads) = 332 reads
- Using the DRS dataset *Alt. prom. A*^*pos*^ *output ≈ intact longFOXP3 ≈ 0.12 X coverage over canon. prom*.
- In the end, the ratio of non-adenylated to adenylated transcripts originating from the upstream promoter can be calculated as *ratio Alt. prom. poly(A)*^*neg*^ *output : Alt. prom. poly(A)*^*pos*^ *output = (NGS coverage over Alt. prom. − 0.12 X NGS* coverage over canon. prom.)/*0.12X NGS* coverage over canon. prom.) *≈ (279–0.12 X 463)/0.12 X 463 ≈ 4*

The coverage values were retrieved from the ‘coverage track’ of IGV when the genome window was centred on the target regions, i.e. the region of probable transcription initiation for the canonical FOXP3 mRNA transcript (chrX:49264670–49264735, NGS coverage: 463) and a region of probable transcription initiation for longFOXP3. The number plotted is the average coverage of a region, which will vary depending on the zoom level you are looking at. The coverage over the alternative promoter region was not homogeneous with each region of probable transcription initiation showing very different coverage values. CAGE peak 1 was considered as the most representative TSS of longFOXP3 in this case (region of probable transcription initiation for peak 1: chrX:49266233–49266279, NGS coverage: 272) to avoid the effect of other CAGE peaks decreasing the mean coverage of the whole promoter region. Of note, we did not observe strand bias upon visual inspection of the individual RNA-Seq reads (i.e. approximately the same number of reads mapped to the template and the complementary strands), which may rule out sequencing artefacts in this region despite the drops in coverage.

### 4.3. In-silico analysis of the coding capacity of longFOXP3

Analysis of the coding capacity of longFOXP3 was done using the software SnapGene Viewer v5.1.2, adjusting the translation options of its ORF finder tool according to the possible and START codons (AUG or CUG) and the minimum length (number of codons). The criteria used for the prediction of microproteins were (i) a minimum length of 150nt including the stop codon, and (ii) only AUG and CUG START codons. The Kozak score for the putative START codons was calculated using the online version of the algorithm *TIS Predictor* [46] (https://www.tispredictor.com/).

### 4.4. Western blot

A usual protocol for both cell lysate preparation and Western blotting was followed. Briefly, 100uL of lysis buffer was added to cell pellets of 1–1.5 X10(6) cells and resuspended by pipetting up-and-down minimizing foam. Mild vortexing was applied and the suspension was incubated on ice for 10 min. Next the lysate was briefly vortexed again and incubated for 10 min at RT. Clearing was done by centrifugation at 13,000 Xg at 4°C for 30 min. The supernatant (protein lysate) was removed without disturbing the pellet and quantified using the BCA assay (BCA protein assay kit, ThermoScientific, ref. # 23227). Approximately 7.5 – 20ug of total protein were seeded in the gels according to availability and cell type. Two PAGE systems were used: (i) Pre-cast 4–12% gradient PAGE. Sample Buffer (4X) and Sample Reducing Agent (10X) were added to the protein lysates and then heated at 70 °C for 10 minutes. Molecular ladder used: Prestained Protein Ladder, 10 to 180 kDa (ii) Homemade standard 12.5% PAGE: Laemmli buffer 2X was added to the protein lysates and then boiled for 5 minutes. Molecular ladder used: Prestained Protein Ladder, 10 to 250 kDa.

The purified monoclonal primary antibodies used to detect FOXP3 were purchased from eBioscience: clone 236A/E7 (mouse, IgG1, k, cat. Number: 14-4777-82), and clone 150D/E4 (mouse, IgG1, k, cat. Number: 14-4774-82). The incubation was done overnight, with rotation, at 4°C and at a 1:1000 dilution. As both primary antibodies were raised in mouse, HRP-conjugated anti-mouse Ig was used as secondary antibody (1–1.5 hours at RT with rotation). However, with PAGE system (i), the secondary antibody was used at 1:20000 (anti-mouse IgG HRP Linked Whole Ab Cytiva NA931-1 ML (Sigma: GENA931), and with PAGE system (ii), the secondary antibody was used at 1:10000 (goat anti-mouse Ig, Human ads-HRP (SouthernBiotech, ref. # 1010–05).

### 4.5. Search for FOXP3 proteoforms and microproteins in public proteomic mass spectrometry dataset

In order to search for protein products of hypothetical microproteins (uORFS) and FOXP3 proteoforms we obtained the raw data of the deepest immune cell proteome available to our knowledge, covering 28 human immune cell types in steady and activated states ([17], EMBL-EBI PRIDE PXD004352). Data were analysed with the MaxQuant software package (v2.0.3.0; [47]) using human data base from UniProt (2022–03, including isoforms) and FASTA file containing sequences of predicted FOXP3 isoforms and uORFs encoded microproteins. Two types of searches were performed, using similar standard search parameters, but different false discovery rate (FDR) cut-offs. The first search was performed using standard FDR cut-off of 1% for peptide and protein identifications. For the second search a strategy adjusted for microprotein identification was applied [48–50]. FDR cut-off for peptides was set to 5% and the FDR cut-off for protein identifications was switch off, because for small proteins only limited numbers of unique peptides are theoretically available and the protein FDR is often overestimated, especially in large proteomics datasets. Common standard parameters were the following: variable modifications of methionine oxidation, deamidation of asparagine and glutamine and N-terminal acetylation, and fixed modification of carbamidomethyl cysteine, minimal peptide length of seven amino acids and a maximum of 3 missed cleavages allowed, match between runs function was activated to allow identification of peptides without MS/MS information. Confident peptide or protein identifications had to fulfil the following criteria: **(i)** minimum of two MS/MS evidences (MS/MS count) per peptide, and **(ii)** If the protein or protein proteoform was detected in more than one replicate, it was considered robustly identified. In addition, MS/MS spectra were manually inspected for peptide sequence coverage.

### 4.6. Flow cytometry

All antibodies used for flow cytometry are detailed in S6 Table. Cells (0.2–1.5 X 10 [5]) were stained in V-bottom 96 multiwell plates. The incubation volume in any staining step was 50ul. When appropriate, the surface staining antibody cocktail included a fixable viability dye. The washing volumes were at least 4-fold the incubation volume (i.e. 250ul). The centrifugation steps consisted in (i) surface staining: 350 Xg, 3 min, at 4°C, and (ii) after fixation: 600 Xg, 3 min, at 4°C. Fixation and permeabilization was done with the eBioscience FOXP3/Transcription factor fixation/Permeabilization kit (Invitrogen) following most of the manufacturer`s instructions, except that: (a) 100ul instead of 200ul of fixation buffer reagent were used, (b) Fixation time was 30 min on ice, and (c) one wash with 200ul of PBE was performed to stop the fixation and wash away the rest of fixation buffer before proceeding to the permeabilization step. Intracellular staining was performed at 4°C for 1 hr. After washing with Permeabilization buffer, another wash with PBE was done before final resuspension in FACS tubes. The acquisition was done on a BD Symphony cell analyzer (BD FACSymphony™ A5 Cell Analyzer) or BD LSR Fortessa cell analyzer (BD LSRFortessa™ Cell Analyzer). Data were analysed on FlowJo software 10.6.1 (TreeStar).

### 4.7. Transfection (electroporation)

The delivery of mRNA or RNPs into cells was done via electroporation with the NEON transfection system in a 10ul format. Most manufacturer`s recommendations were followed, except that (i) T-buffer was used to transfect cell lines instead of the recommended R-buffer provided with the kit, and (ii) after electroporation cells were directly pipetted into Antibiotic-supplemented medium. Briefly, cells were harvested, pelleted (350×g, 5 min, at 20°C), resuspended in PBE buffer at RT and counted. Cell pellets were resuspended in T-buffer to a final concentration of ~ 5.6–6.7 X10(7)/mL. Nine (9) uL of the cell suspension (5–6 X 10(5) cells) were then pipetted onto 2.5uL of payload (RNP or mRNA diluted in T-buffer) for a final volume of 11.5uL, and 10 uL were used per electroporation. The same electroporation settings were used in every case: Amplitude: 1600 V/Pulse width: 10msec/Number of pulses: 3. After the electric shock, the cells were pipetted into either 200ul of culture medium in a 96-mwell plate (RNP-KO experiments) or 500ul of culture medium in a 48-mwell plate (Over-expression assay).

### 4.8. mRNA synthesis by *in vitro* transcription (IVT)

Importantly, no silent mutations were introduced for the generation of the IVT mRNA templates to avoid creating or destroying *cis*-regulatory elements. The generation of IVT mRNA consists in 4 major steps. Namely: (1) Preparation of the DNA template by PCR, (2) *In vitro* transcription, (3) DNase treatment, and (4) mRNA Purification. Briefly, the PCR reaction was carried out using NEB High Fidelity kit to minimize the introduction of mutations. The reaction was set to 50ul, and 1uM Forward and Reverse primers were used together with 1 ng of DNA template. Different plasmid vectors (a) pRNA-(A)128_GFP, b) pRNA-(A)128_canonical FOXP3), or c) pRNA-(A)128_longFOXP3)) were used as template for different IVT products. The amplicons were cleaned up by running the whole PCR reaction in a 1% agarose gel (at 100 V, for 1 hour), cutting the bands of expected size, followed by gel purification (gel extraction kit MN, Germany). The concentration of the purified fragment was measured using UV/Vis-spectroscopy (NanoDrop 1000 Spectrophotometer; PEQLAB). The mRNAs were synthesized using 1ug of PCR amplicon and the Transcript Aid T7 high Yield Transcription Kit following the manufacturer’s instruction. The 5′ end of mRNA was modified co-transcriptionally with anti – reverse cap analog (ARCA) (Jena Bioscience, Germany). No chemical modifications were incorporated to IVT products. Next the DNA template was removed via incubation with DNaseI at 37°C, for 15 min. Finally, the IVT products were purified by standard lithium chloride-based precipitation. DEPC-treated RNase free water and lithium chloride (LiCl) were added to the mRNA products to the final concentration of 2.5 M and the reaction was incubated at − 20 °C overnight followed by centrifugation at 13,000 g at 4 °C for 30 minutes. Further washing was done using 70 vol% cold ethanol, and final mRNA products were resuspended in FACSymphony™ nuclease-free sterile water (Merck Millipore, Germany). All IVT-mRNAs were analysed by denaturing agarose gel electrophoresis for integrity and homogeneity and the concentration was determined photospectroscopically.

### 4.9. Over-expression assay

Importantly, the size of the longFOXP3 construct made it impossible to have approximately similar amount of total RNA transfected per condition if equal number of copies were intended. Therefore, equimolar amounts of the canonical and longFOXP3 transcripts were used while ensuring that the amount of RNA payload was not toxic (S7 Fig). Priority to normalization to IVT transcript copy number was given and RNA payload was NOT higher than ~ 550ng/5–6 X10 [5] cells) to prevent any toxic effect across any cell type. In all experiments, four conditions were tested: condition 1) cell were mock-transfected; condition 2) cells were transfected only with GFP mRNA; condition 3) cells were transfected with a mixture of GFP mRNA and the canonical FOXP3 construct; and condition 4) cells were transfected with GFP mRNA and the longFOXP3 transcript. The readout was done at 8hs post-transfection and divided in two steps: 1) FACS analysis of the fluorescent reporter levels to account for delivery and translation competence of the recipients 2) Full standard protocol for FOXP3 detection. Two parameters were analysed: (i) fold change of the percentage of FOXP3+ cells and (ii) fold change of MFI(FOXP3) in the living singlet gate. The values for both parameters were normalized to allow comparison of cells transfected with the canonical FOXP3 IVT or the longFOXP3 construct. To this end, the values were compared to those values in cells transfected with IVT GFP mRNA only. Percentage of GFP+ cells was chosen as normalizer because the MFI(GFP) showed higher standard deviation across all conditions and cell types. To normalize the fold change in percentage of FOXP3+ cells the following equation was used [(% FOXP3+ in condition 3 or 4/% FOXP3+ in condition 2)/(% GFP+ in condition 3 or 4/% GFP+ in condition 2)]. Similarly, to normalize the fold change in MFI(FOXP3) the following equation was employed [(MFI(FOXP3) in condition 3 or 4/MFI(FOXP3) in condition 2)/(% GFP+ in condition 3 or 4/% GFP+ in condition 2)].

### 4.10. CRISPR-Cas9-mediated protein knock-out

Guide RNAs (gRNAs) were designed using different algorithms or taken from previous publications (S7 table), and purchased from Synthego Corporation in a single guide format (sgRNAs). SgRNAs were *in vitro*-assembled with the Cas9 protein (Alt-R™ S.p. Cas9-RFP V3, IDT) to form the effector ribonucleoproteins (RNPs). The sgRNA and Cas9 protein were incubated for 15–30 minutes at RT while preparing the cells for the electroporation. RNPs were used at a final concentration of 1–1.5 uM. Delivery efficiency was assessed by detection of RFP signal by FACS analysis 24 and 48hs after transfection. TotalFOXP3 knock-out efficiency was only assessed by FACS analysis of the resulting samples four days after the electroporation and after 24 hours of restimulation with T_REG_ expansion beads at 1:1 ratio.

### 4.11. Prime flow assay

The PrimeFlow assay is an RNA-FISH technique that achieves single-molecule detection sensitivity due to its amplification strategy, which relies on serial hybridization steps, and employs a probe design strategy that also secures high specificity. Figure 4A shows a simplified scheme of how the technology works and of the targets we expected to detect with our experimental design. Two probe sets were used: the *UPA_2 probe* set targeted the majority of the extended 5´-UTR, whereas the *total FOXP3 mRNA probe* was directed to a shared sequence between the full-length and delta2 splice variants of the canonical and longFOXP3 transcripts (S10 Fig). An anti-human FOXP3 monoclonal antibody capable of detecting all FOXP3 proteoforms was also included in the panel.

Human T_REG_ cultures established from either naive-like or truly naive T_REGS_ (nT_REG_ as grouping name) were analysed. Furthermore, T_CONV_ and in vitro-induced T_REG_ (iT_REG_) cultures were used as biological controls. The latter are T_CONV_ cells in which the expression of FOXP3 is enforced by TCR stimulation in the presence of IL-2 and TGF-B1, but these cells do not acquire a stable T_REG_ identity [51]. Expanded nT_REG_ cells were reactivated under normal culture conditions for approximately eight days before the readout. In parallel, naive-like T_CONV_ cultures were reactivated and treated either with recombinant human Interleukin-2 (rhIL-2, 100IU/mL) or with a cocktail of rhIL-2 (100IU/mL) and recombinant human transforming growth factor beta-1 (rhTGFβ-1, 5 ng/mL) (iT_REG_ conditions) to work as biological negative controls for detection with the UPA_2 probe.

The protocol for staining described in the PrimeFlow user guide provided by Thermo Fischer Scientific was strictly followed (PrimeFlow™ RNA Assay Kit USER GUIDE [16]). Incubation at 40°C was done in a normal incubator (Inkubationshaube TH 15, Edmund Bühler GmbH) and the temperature was monitored using a thermometer left inside at all time. No fewer than 1 X 10(6) and up to 5 X 10(6) cells/well were stained. The staining was done on a V-bottom 96-multiwell plate (plate format). After the final wash, the cells were resuspended in 250 – 300ul PBE and transferred into a FACS tube.

### 4.12. Confocal microscopy

#### 4.12.1. Rnascope assay sample preparation

The principle of the RNAscope assay is the same as that of the PrimeFlow assay and has been described elsewhere [52]. We applied a two-probe approach to detect target RNAs while using DAPI counterstaining to detect nuclei. The *total FOXP3 mRNA* probe was the same as in the PrimeFlow assay while the probe to detect the upstream transcripts (*UPA_Scope*) was redesigned to exclude any sequence shared with FLICR and avoid any contribution of this potential confounding factor (S10 Fig). Roughly, each speckle was interpreted as one transcript and no discrimination was made in terms of the size or intensity of the individual spots. Their subcellular localization was defined as either nuclear (by co-localization with DAPI) or cytoplasmic. See S11 Fig. for a more detailed explanation.

Four primary T cell subsets were analysed: naive-like T_CONV_ (called naive T_CONV_), memory T_CONV_, truly naive T_REG_ (called naive T_REG_), and memory T_REG_. The analysis was performed in four healthy donors processed in batches of two. After six to seven days of culture primary T cells were treated according to the protocol provided by Advanced Cell Diagnostics (ACD) [53] to prepare PBMCs and non-adherent cells for the FACSymphony™ assay. After the cells were fixed and washed, they were resuspended in EtOH 70% at 1 X10(6) cell/ml, and 10 µl were pipetted inside wells generated by attaching a small IBIDI insert of a glass slide. After centrifugation (500 Xg, 3 min, RT), the supernatant was gently removed and the subsequent steps of the slide preparation were followed. After the last dehydration step with EtOH 100%, the slides were stored overnight at −20°C. On the next day the IBIDI inset was removed and the RNAscope staining was done following the instructions detailed in the LSRFortessa™ Fluorescent Multiplex Kit User Manual [54]. Samples were incubated with the same probe cocktail directed to the target RNAs: UPA_Scope probe and totalFOXP3 mRNA probe. In the first experiment, a spare set of samples was incubated with a universal negative control directed against bacterial DapB RNA (provided with the kit) to assess unspecific signal. The cells were then incubated with DAPI solution (provided with the kit) for 2 minutes and washed. Finally, the slides were mounted using ProLong Gold antifade mounting medium (ThermoFisher Scientific) and kept at 4°C in the dark until acquisition on the confocal microscope.

#### 4.12.2. Confocal image acquisition

All RNAScope stainings were acquired at high resolution (104 nm) with a Zeiss LSM-880 confocal microscope using a 63× objective. All microscope settings including laser power stayed constant throughout the experiments. The images were processed and analysed with CellProfiler software following Nature’s guide for digital images when applicable.

#### 4.12.3. Fully automated counting of fluorescent foci (speckle), co-localization and subcellular localization analysis

Images were preprocessed for optimal segmentation by manually adjusting the Intensities of the respective staining to achieve high signal-to-noise ratio. A custom-made pipeline in CellProfiler 3.1.819. was used for automated analysis. Pipelines (.cpproj files) and confocal microscopy images used as input (.tiff files) are accessible in S3 file.

In short, by applying the *IdentifyPrimaryObjects* module to the DAPI input image, nuclei were defined as primary objects. Cells were identified by expansion of the nuclei objects by 3 µm (29 pixels) representing the approximate size of the cytoplasm in T cells, and cytoplasm objects were defined by subtracting the nuclei objects from the respective cells objects. As we did not intend to compare intensities but aimed to achieve optimal segmentation, if necessary, the intensity of the input images of *total FOXP3 mRNA* and *UPA_Scope* was adapted in order to get a high signal-to-noise ratio for optimal subsequent spot detection. *Total FOXP3 mRNA* and *UPA_Scope* speckles were segmented by applying the IdentifyPrimaryObjects to the optimized image of *total FOXP3 mRNA* and *UPA_Scope*. Identified spots of *total FOXP3 mRNA* and *UPA_Scope* were each masked to both, the cell objects and the nuclei objects to count the number of spots per cell and nuclei for each object. Co-localization was measured by using the module *MaskObjects* applied on identified *UPA_Scope* and *total FOXP3 mRNA* objects with a required overlap of 0.3. The value 0.3 was chosen by intensive testing of different parameters and visual inspection of correct co-localization, and subsequently applied to all analysed images. Results included the objects of identified cells and nuclei, the number of *total FOXP3 mRNA* objects, *UPA_Scope* objects, and co-localizing spot objects per cell and nuclei, and were exported as csv files. Subsequent data tidying, analysis and visualization was performed using R and RStudio.

#### 4.12.4. Statistical analysis

To compare the proportion of cells that contained speckles and because the number of cells analysed varied widely across T cell subsets (conditions) and donors (range: 140–1390) (S2–3 Table), we used a logistic regression (LR)-based approach to analyse the changes of proportions across groups, as previously discussed by Douma *et al*. 2019 [55]. LR analysis methods (and generalized linear models – GLM-) can be used to test contingency tables of counts from which proportions are derived [56]. Therefore, we conducted an LR-based analysis to test the change in the fraction of cells displaying total FOXP3 mRNA speckles or UPA_Scope speckles between any two conditions (e.g. naive T_CONV_ vs memory T_CONV_) by testing the significance of the regression coefficient. We repeated this analysis for each pair of cell types and we corrected the p-values across pairwise tests using Bonferroni´s multiple comparisons correction method [57]. Furthermore, we used the biological replica information as a covariate in the LR model to remove the confounding effect caused by the differences between donors.

Specifically, to test the changes in the proportion of cells with total FOXP3 mRNA or UPA speckles (target speckles) across conditions we used the ‘glm’ function in R with the family = ‘binomial’ option to test the changes in the log odds ratio of cells with target speckles given the condition (cell type). To apply the ‘glm’ function, we transformed the contingency tables as a large data table with three columns: ‘Donor’, ‘CellType’ and ‘Empty’, where each row describes the donor/replicate of origin of a cell, the cell type and whether the cell has at least one speckle or not (see S3 files). Here, each cell is an individual data point where (i) the response variable in the logistic regression model is 0=Empty (no speckle) and 1=NonEmpty (at least one target speckle), and (ii) input variable of the model is the cell type posed as a binary categorical variable. We used the biological replicate (healthy donor) of origin of each cell as a covariate term for the logistic regression model to regress out the donor effect. Finally, we used the ‘p.adjust’ function in R with method = ‘bonferroni’ argument to adjust p-values associated with the significance of regression coefficients across multiple logistic regression models (each regression model is associated with a pair of conditions, i.e. cell type).

### 4.13. Data analysis and statistics

Except for the analysis of the RNAscope dataset, statistical analysis were performed using GraphPad Prism Version 9.4.1 (GraphPad software) in the following cases: (a) to compare two independent populations, a non-parametric unpaired Mann-Whitney test was used, (b) to compare the effect of over-expressing the canonical FOXP3 and longFOXP3 transcripts, a non-parametric paired Wilcoxon test was performed. P-values <0.05 were considered significant. Statistically significant differences were always shown on the plots except for Figure 1D, in which non-significance is detailed, and for Figure 5C,D, in which statistically significance was only mentioned in the text.

## Supplementary Material

Manuscript_RNA Biology_Supporting information.docx

## Data Availability

All nanopore DRS (fast5/pod5) and Illumina datasets (fastq) are available at the European Nucleotide Archive/Sequence Read Archive under the accession/project number (PRJEB81485).

## List of abbreviations

AA or aa.: Aminoacid
CAGE: Cap Analysis of Gene Expression
CAT: CAGE Associated Transcriptome
CDS: Coding Sequence = ORF = Open Reading Frame.
DRS: direct (or native) RNA sequencing
FANTOM: Functional Annotation of Mammalian Genome
FLICR: FOXP3 Regulating Long Intergenic Non-Coding RNA
FOXP3: Forkhead Box Protein 3
IGV: Integrated genome Viewer (genome browser tool)
rh-IL: recombinant human Interleukin
iT_REG_: in-vitro-induced T_REG_
IVT mRNA: *in vitro*-transcribed messenger RNA
lncRNA: long non-coding RNA
Mpro: Microprotein
MW: Molecular Weight
nT_REG_: natural T_REG_
NRCeq: Nanopore ReCappable sequencing
ORF: Open Reading Frame
PAM: Protospacer Adjacent Motif
PAS: Polyadenylation Signal
PBMC: Peripheral Blood Mononuclear Cell
RACE: Rapid Analysis of cDNA Ends
smFISH: single-molecule fluorescence *in vitro* hybridization
T_CONV_: conventional T cell
TERA-seq: True-end-to-end RNA sequencing
TIS: Translation Initiation Site
T_REG_: Regulatory T cell
TSCM: Stem cell-line memory T cell
TSS: Transcription Start Site
uORF: Upstream ORF
UTR: Untranslated Region

## Code availability

Scripts for generating figures and the logistic regression based contingency table test can be found in https://github.com/mikrohscopist/Spot_colocalization_analysis.

## References

[1] Zemmour D, Pratama A, Loughhead SM, et al. Flicr, a long noncoding RNA, modulates Foxp3 expression and autoimmunity. Proc Natl Acad Sci USA. 2017;114(17):E3472–E80. doi: 10.1073/pnas.1700946114 Epub 20170410. 28396406 PMC5410798

[2] Ono M. Control of regulatory T-cell differentiation and function by T-cell receptor signalling and Foxp3 transcription factor complexes. Immunology. 2020;160(1):24–37. doi: 10.1111/imm.13178 Epub 20200309. 32022254 PMC7160660

[3] Colamatteo A, Carbone F, Bruzzaniti S, et al. Molecular mechanisms controlling Foxp3 expression in health and autoimmunity: from epigenetic to post-translational regulation. Front Immunol. 2019;10:3136. doi: 10.3389/fimmu.2019.03136 Epub 20200203. 32117202 PMC7008726

[4] Du J, Wang Q, Yang S, et al. FOXP3 exon 2 controls T(reg) stability and autoimmunity. Sci Immunol. 2022;7(72):eabo5407. doi: 10.1126/sciimmunol.abo5407 Epub 20220624. 35749515 PMC9333337

[5] Robinson EK, Jagannatha P, Covarrubias S, et al. Inflammation drives alternative first exon usage to regulate immune genes including a novel iron-regulated isoform of Aim2. Elife. 2021;10. doi: 10.7554/eLife.69431 Epub 20210528. PMC8260223 34047695

[6] Rubtsov PM. Alternative promoters and RNA processing in expression of the eukaryotic genome. Mol Biol (Mosk). 2000;34(4):626–634. doi: 10.1007/BF02759562 11042854

[7] Mitschka S, Mayr C. Context-specific regulation and function of mRNA alternative polyadenylation. Nat Rev Mol Cell Biol. 2022;23(12):779–796. doi: 10.1038/s41580-022-00507-5 Epub 20220707. 35798852 PMC9261900

[8] Lewis ZA. Expanding the proteome: A-to-I RNA editing provides an adaptive advantage. Proc Natl Acad Sci USA. 2023;120(16):e2303563120. doi: 10.1073/pnas.2303563120 Epub 20230410. 37036963 PMC10120046

[9] Davuluri RV, Suzuki Y, Sugano S, et al. The functional consequences of alternative promoter use in mammalian genomes. Trends Genet. 2008;24(4):167–177. doi: 10.1016/j.tig.2008.01.008 Epub 20080307. 18329129

[10] Singer GA, Wu J, Yan P, et al. Genome-wide analysis of alternative promoters of human genes using a custom promoter tiling array. BMC Genomics. 2008;9(1):349. doi: 10.1186/1471-2164-9-349 Epub 20080725. 18655706 PMC2527337

[11] Schmidl C, Hansmann L, Lassmann T, et al. The enhancer and promoter landscape of human regulatory and conventional T-cell subpopulations. Blood. 2014;123(17):e68–78. doi: 10.1182/blood-2013-02-486944 Epub 20140326. 24671953

[12] Eckerstorfer P, Novy M, Burgstaller-Muehlbacher S, et al. Proximal human FOXP3 promoter transactivated by NF-kappaB and negatively controlled by feedback loop and SP3. Mol Immunol. 2010;47(11–12):2094–2102. doi: 10.1016/j.molimm.2010.04.002 Epub 20100511. 20462637 PMC6340486

[13] Poliseno L, Lanza M, Pandolfi PP. Coding, or non-coding, that is the question. Cell Res. 2024;34(9):609–629. doi: 10.1038/s41422-024-00975-8 Epub 20240725. 39054345 PMC11369213

[14] Passmore LA, Coller J. Roles of mRNA poly(A) tails in regulation of eukaryotic gene expression. Nat Rev Mol Cell Biol. 2022;23(2):93–106. doi: 10.1038/s41580-021-00417-y Epub 20210930. 34594027 PMC7614307

[15] Andreev DE, Loughran G, Fedorova AD, et al. Non-AUG translation initiation in mammals. Genome Biol. 2022;23(1):111. doi: 10.1186/s13059-022-02674-2 Epub 20220509. 35534899 PMC9082881

[16] Scientific TF. PrimeFlow™ RNA assay kit user guide. 2022.

[17] Rieckmann JC, Geiger R, Hornburg D, et al. Social network architecture of human immune cells unveiled by quantitative proteomics. Nat Immunol. 2017;18(5):583–593. doi: 10.1038/ni.3693 Epub 20170306. 28263321

[18] Volta V, Perez-Baos S, de la Parra C, et al. A DAP5/eIF3d alternate mRNA translation mechanism promotes differentiation and immune suppression by human regulatory T cells. Nat Commun. 2021;12(1):6979. doi: 10.1038/s41467-021-27087-w Epub 20211130. 34848685 PMC8632918

[19] Shuman S. Transcriptional interference at tandem lncRNA and protein-coding genes: an emerging theme in regulation of cellular nutrient homeostasis. Nucleic Acids Res. 2020;48(15):8243–8254. doi: 10.1093/nar/gkaa630 32720681 PMC7470944

[20] Yang L, Duff MO, Graveley BR, et al. Genomewide characterization of non-polyadenylated RNAs. Genome Biol. 2011;12(2):R16. doi: 10.1186/gb-2011-12-2-r16 Epub 20110216. 21324177 PMC3188798

[21] Calvo SE, Pagliarini DJ, Mootha VK. Upstream open reading frames cause widespread reduction of protein expression and are polymorphic among humans. Proc Natl Acad Sci USA. 2009;106(18):7507–7512. doi: 10.1073/pnas.0810916106 Epub 20090416. 19372376 PMC2669787

[22] Jia L, Mao Y, Ji Q, et al. Decoding mRNA translatability and stability from the 5’ UTR. Nat Struct Mol Biol. 2020;27(9):814–821. doi: 10.1038/s41594-020-0465-x Epub 20200727. 32719458

[23] Lin D, Hiron TK, O’Callaghan CA. Intragenic transcriptional interference regulates the human immune ligand MICA. Embo J. 2018;37(10). doi: 10.15252/embj.201797138 Epub 20180411. PMC5978299 29643123

[24] Tsuchida CA, Brandes N, Bueno R, et al. Mitigation of chromosome loss in clinical CRISPR-Cas9-engineered T cells. Cell. 2023;186(21):4567–82 e20. doi: 10.1016/j.cell.2023.08.041 Epub 20231003. 37794590 PMC10664023

[25] Ghavami S, Pandi A. CRISPR interference and its applications. Prog Mol Biol Transl Sci. 2021;180:123–140. doi: 10.1016/bs.pmbts.2021.01.007 Epub 20210212. 33934834

[26] Taiana E, Favasuli V, Ronchetti D, et al. In vitro silencing of lncRNAs using LNA GapmeRs. Methods Mol Biol. 2021;2348:157–166. doi: 10.1007/978-1-0716-1581-2_10 34160805

[27] Brunkow ME, Jeffery EW, Hjerrild KA, et al. Disruption of a new forkhead/winged-helix protein, scurfin, results in the fatal lymphoproliferative disorder of the scurfy mouse. Nat Genet. 2001;27(1):68–73. doi: 10.1038/83784 11138001

[28] Hollerer I, Barker JC, Jorgensen V, et al. Evidence for an integrated gene repression mechanism based on mRNA isoform toggling in human cells. G3 (Bethesda). 2019;9(4):1045–1053. doi: 10.1534/g3.118.200802 Epub 20190409. 30723103 PMC6469420

[29] Childs-Disney JL, Yang X, Gibaut QMR, et al. Targeting RNA structures with small molecules. Nat Rev Drug Discov. 2022;21(10):736–762. doi: 10.1038/s41573-022-00521-4 Epub 20220808. 35941229 PMC9360655

[30] Revenko A, Carnevalli LS, Sinclair C, et al. Direct targeting of FOXP3 in Tregs with AZD8701, a novel antisense oligonucleotide to relieve immunosuppression in cancer. J Immunother Cancer. 2022;10(4):e003892. doi: 10.1136/jitc-2021-003892 35387780 PMC8987763

[31] Ibrahim F, Oppelt J, Maragkakis M, et al. TERA-Seq: true end-to-end sequencing of native RNA molecules for transcriptome characterization. Nucleic Acids Res. 2021;49(20):e115. doi: 10.1093/nar/gkab713 34428294 PMC8599856

[32] Ugolini C, Mulroney L, Leger A, et al. Nanopore ReCappable sequencing maps SARS-CoV-2 5’ capping sites and provides new insights into the structure of sgRNAs. Nucleic Acids Res. 2022;50(6):3475–3489. doi: 10.1093/nar/gkac144 35244721 PMC8989550

[33] Mulroney L, Wulf MG, Schildkraut I, et al. Identification of high-confidence human poly(A) RNA isoform scaffolds using nanopore sequencing. RNA. 2022;28(2):162–176. doi: 10.1261/rna.078703.121 Epub 20211102. 34728536 PMC8906549

[34] Thompson SR. So you want to know if your message has an IRES? Wiley Interdiscip Rev RNA. 2012;3(5):697–705. doi: 10.1002/wrna.1129 Epub 20120625. 22733589 PMC3419317

[35] Li H, Birol I. Minimap2: pairwise alignment for nucleotide sequences. Bioinformatics. 2018;34(18):3094–3100. doi: 10.1093/bioinformatics/bty191 29750242 PMC6137996

[36] Li H, Handsaker B, Wysoker A, et al. The sequence alignment/map format and SAMtools. Bioinformatics. 2009;25(16):2078–2079. doi: 10.1093/bioinformatics/btp352 Epub 20090608. 19505943 PMC2723002

[37] Robinson JT, Thorvaldsdottir H, Winckler W, et al. Integrative genomics viewer. Nat Biotechnol. 2011;29(1):24–26. doi: 10.1038/nbt.1754 21221095 PMC3346182

[38] Bushnell B, editor. BBMap: A fast, accurate, Splice-Aware Aligner. In: Bushnell B, editor. Conference: 9th annual genomics of energy & environment meeting. Walnut Creek, CA; 2014 Mar 17–20.

[39] Team RC. R: a language and environment for statistical computing. Vienna: R Foundation for Statistical Computing; 2023. https://www.R-project.org/

[40] team P. Rstudio: integrated development environment for R. Posit. Posit Softw, PBC. 2024.

[41] Barrett TD, Srinivasan A, Gorecki J, et al. Data.Table: extension of ‘data.Frame’. version 1.15.4 ed2024. p. R package.

[42] Wickham HF, Henry L, Müller K. Vaughan D dplyr: a grammar of data manipulation. version 1.1.4 ed2023. p. R package. 2025. https://github.com/tidyverse/dplyr

[43] Wickham H. ggplot2: elegant graphics for data analysis. New York: Springer-Verlag; 2016.

[44] P T. Patchwork: the composer of plots. version 1.2.0 ed2024. p. R package.

[45] Dobin A, Davis CA, Schlesinger F, et al. STAR: ultrafast universal RNA-seq aligner. Bioinformatics. 2013;29(1):15–21. doi: 10.1093/bioinformatics/bts635 Epub 20121025. 23104886 PMC3530905

[46] Gleason AC, Ghadge G, Chen J, et al. Machine learning predicts translation initiation sites in neurologic diseases with nucleotide repeat expansions. PLOS ONE. 2022;17(6):e0256411. doi: 10.1371/journal.pone.0256411 Epub 20220601. 35648796 PMC9159584

[47] Cox J, Neuhauser N, Michalski A, et al. Andromeda: a peptide search engine integrated into the MaxQuant environment. J Proteome Res. 2011;10(4):1794–1805. doi: 10.1021/pr101065j Epub 20110222. 21254760

[48] van Heesch S, Witte F, Schneider-Lunitz V, et al. The translational landscape of the human heart. Cell. 2019;178(1):242–60 e29. doi: 10.1016/j.cell.2019.05.010 Epub 20190530. 31155234

[49] Ma J, Saghatelian A, Shokhirev MN, et al. The influence of transcript assembly on the proteogenomics discovery of microproteins. PLOS ONE. 2018;13(3):e0194518. doi: 10.1371/journal.pone.0194518 Epub 20180327. 29584760 PMC5870951

[50] Mackowiak SD, Zauber H, Bielow C, et al. Extensive identification and analysis of conserved small ORFs in animals. Genome Biol. 2015;16(1):179. doi: 10.1186/s13059-015-0742-x Epub 20150914. 26364619 PMC4568590

[51] Mikami N, Kawakami R, Chen KY, et al. Epigenetic conversion of conventional T cells into regulatory T cells by CD28 signal deprivation. Proc Natl Acad Sci USA. 2020;117(22):12258–12268. doi: 10.1073/pnas.1922600117 Epub 20200515. 32414925 PMC7275710

[52] Wang F, Flanagan J, Su N, et al. Rnascope: a novel in situ RNA analysis platform for formalin-fixed, paraffin-embedded tissues. J Mol Diagn. 2012;14(1):22–29. doi: 10.1016/j.jmoldx.2011.08.002 22166544 PMC3338343

[53] Advanced Cell Diagnostics I. Rnascope® fluorescent assay for PBMC and Non-adherent cells. 2014. Available from: www.acdbio.com/support

[54] Advanced Cell Diagnostics I. Rnascope multiplex fluorescent reagent kit v2 user manual. Available from: www.acdbio.com2022

[55] Douma JC, Weedon JT, Warton D. Analysing continuous proportions in ecology and evolution: a practical introduction to beta and Dirichlet regression. Methods Ecol Evol. 2019;10(9):1412–1430. doi: 10.1111/2041-210X.13234

[56] Nelder JA. Log linear models for contingency tables: a generalization of classical least squares. J R Stat Soc Ser C: Appl Stat. 2018;23(3):323–329. doi: 10.2307/2347125

[57] Goeman JJ, Solari A. Multiple hypothesis testing in genomics. Stat Med. 2014;33(11):1946–1978. doi: 10.1002/sim.6082 Epub 20140108. 24399688

