# Supplementary material for: Novel non-coding FOXP3 transcript isoform associated to potential transcriptional interference in human regulatory T cells
Source: RNA Biol. 2025 Jul 2;22(1):1–20. doi: 10.1080/15476286.2025.2502719 (PMC12233831; doi:10.1080/15476286.2025.2502719)
Supplement: Manuscript_RNA Biology_Supporting information.docx [file KRNB_A_2502719_SM6789.docx]

# Supporting information

# Supporting figures

S1 Fig.


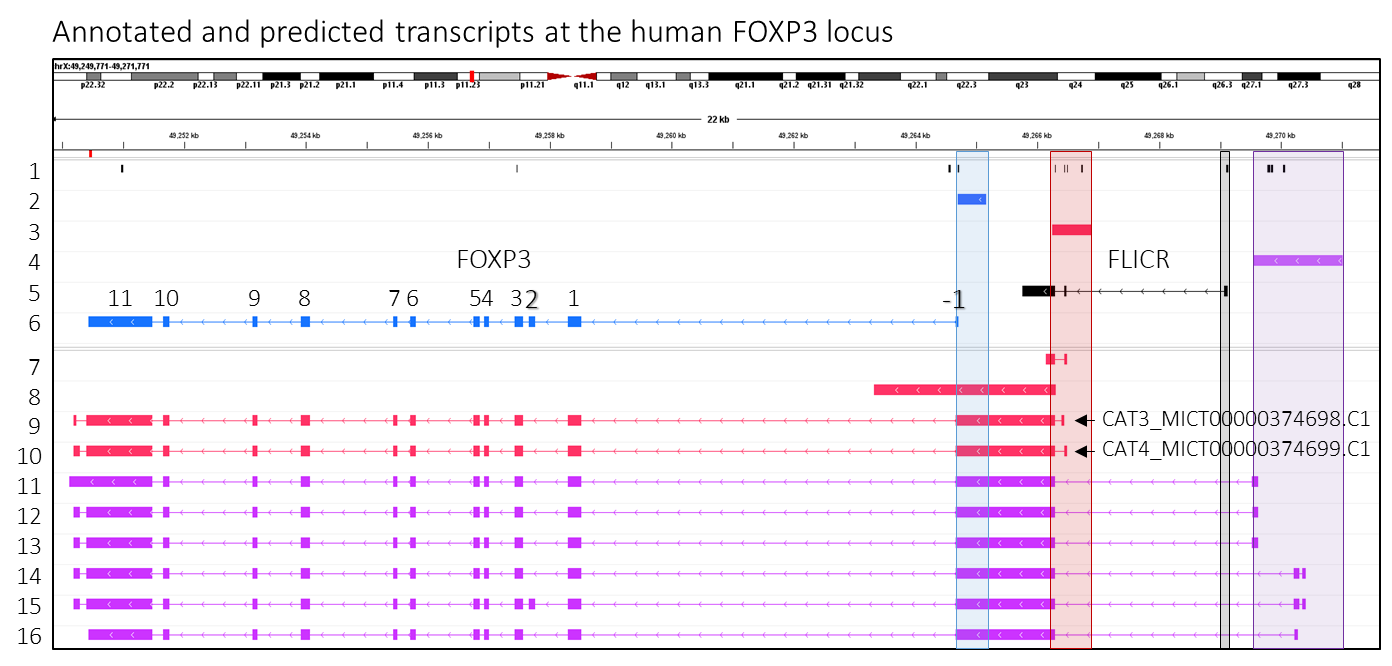


**S1 Fig. The transcriptional output of the human FOXP3 locus is expected to be richer than that described in the current annotation**. IGV browser screenshot of the region encompassing the human FOXP3 and FLICR genes. Track 1 includes robust CAGE peaks from the FANTOM5 consortium. Tracks 2 and 3 include the sequences used as canonical and novel promoters, respectively, in the reporter assays by Schmidl *et al.* (2014). Track 4 includes the sequence used as promoter region in the reporter assays by Eckerstorfer *et al*. (2010). FLICR (Foxp3 long intergenic noncoding RNA) variant 3 (track 5) and the full-length FOXP3 mRNA isoform (track 6) are included as a reference to which the predicted transcripts can be compared against. In humans, FOXP3 is coded on the negative strand of the X chromosome, and therefore, transcripts extend from right to left. The human FOXP3 gene consists of 12 exons (exons -1 – 11) and is mostly transcribed into two mRNA variants: the isoform in which all exons are retained (full-length FOXP3) and the splice variant in which exon 2 is left out (delta2/∆2 FOXP3). FLICR gives rise to three transcript variants (isoforms 1 – 3), but they all share the same TSS and last exon. Tracks 7 – 16 display a selection of transcripts from the FANTOM6 CAT catalogue developed and supported by RIKEN Institute (<https://fantom.gsc.riken.jp/cat/v1/>). Transcripts are color-coded according to the promoter region expected to drive their expression. *By definition, each transcript is associated with one gene and one promoter*. The output of the novel T_REG_-exclusive promoter (red highlight) is expected to be very rich, rendering transcripts that extend across the whole FOXP3 gene (tracks 9 and 10) or not (tracks 7 and 8). Transcript ENCT00000477108.C1 (track 8) is predicted to end within the first intron of FOXP3, ~1.4kb downstream of the canonical FOXP3 TSS. Note that no data are available on whether these RNAs are expected to be polyadenylated.

S2 Fig.


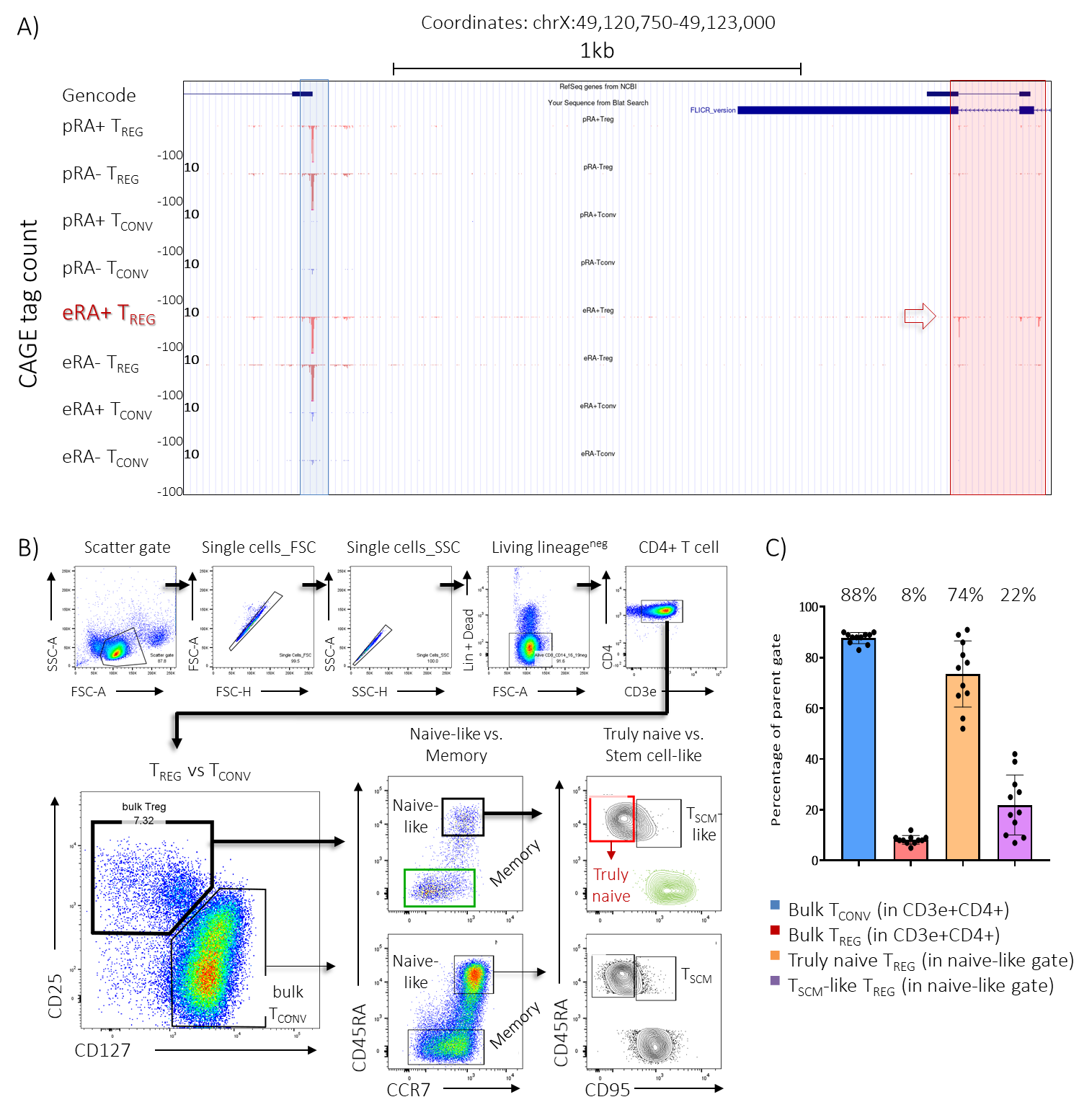


**S2 Fig.** **Description of the T_REG_ compartment in human peripheral blood and choice of target population for DRS experiment. A)** *Adapted from Schmidl et al. (2014)*. UCSC Genome browser screenshot of the DNA stretch encompassed by the canonical and alternative promoters (blue and red transversal highlights, respectively). Each track includes FANTOM5 CAGE data from Schmidl *et al*. (2014) for one of the eight types of samples analyzed (naive or memory conventional and naive or memory regulatory T cell subsets before and after *in vitro* culture). The sample ID is written on the left of each track: “RA+” means that the cells were FACS-sorted as positive for CD45RA (and therefore, considered as naive), “RA-“ means that the sorted cells were negative for CD45RA, “p” stands for ex vivo, while “e” stands for cultured. The data suggested that the alternative promoter was more active in naive T_REGS_ after culture compared to all other samples and conditions analyzed (highlighted by red arrow). **B)** Gating strategy employed to FACS sort human truly naive T_REG_ cells from PBMCs. The naive fraction of peripheral blood-derived T_REGS_ is heterogeneous. Despite the expression of markers typically associated with naive cells, ~22% of CD45RA^hi^CCR7^+^ T_REGS_ expressed the activation/memory marker CD95 (S2C Fig). This is reminiscent of stem cell-like memory T cells (T_SCM_) (1) and is in line with a recent report (2). The whole memory fraction (CD45RA^neg^) of the bulk T_REG_ gate is overlaid on the “Truly naive vs. Stem cell-like” contour plot as a reference. Lin, lineage (CD8a, CD14, CD16, CD19); T_SCM_, Stem cell memory T cell; HD, healthy donor). **C)** Frequencies of different T cell subsets in human peripheral blood. The mean percentage is written on top of each column and the whiskers indicate SD.

S3 Fig.
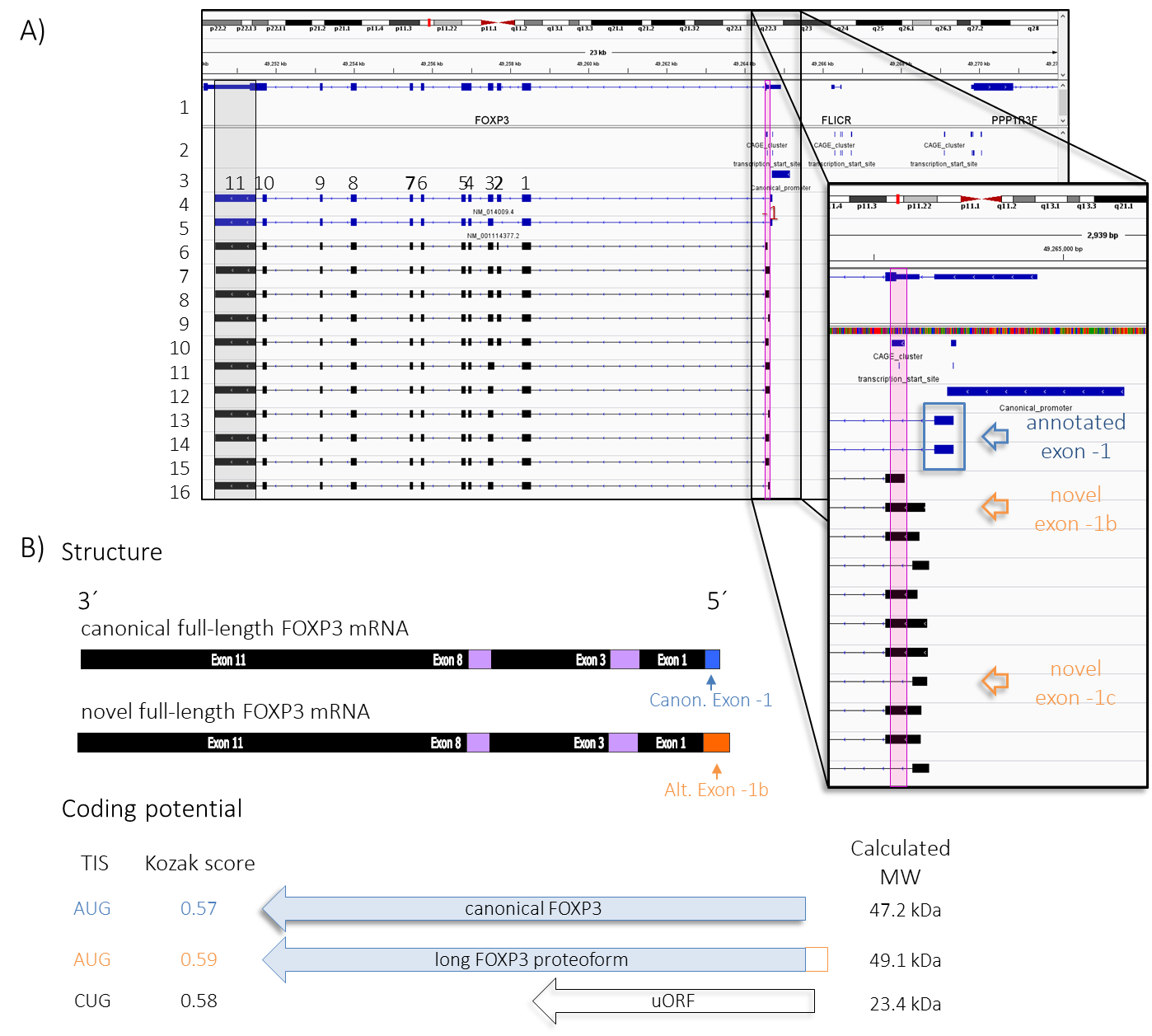


**S3 Fig.** **The canonical promoter gives rise to a novel mRNA isoform with the potential to code for a FOXP3 proteoform with an extended N-terminal domain.** **A)** IGV browser screenshot of the human FOXP3 locus. Track 1 is the RefSeq Genes track, which shows annotated coding and non-coding genes taken from the NCBI RNA reference sequences collection. Track 2 displays FANTOM5 CAGE peaks. For visual reference, the sequence considered as canonical promoter by Schmidl *et al*. (2014) and the canonical full-length FOXP3 and ∆2 FOXP3 mRNA isoforms are included in tracks 4, 5, and 6, respectively. The outset is a zoom-in into the area defined by the black rectangle that encompasses the two CAGE peaks associated with the canonical FOXP3 promoter. Tracks 6 – 17 display a selection of DRS reads obtained from HD1 and HD2. These transcripts start at the CAGE peak with lowest score (*CAGE cluster* hg38::*chrX:49264549-49264582 – purple highlight*), which is closely downstream of the annotated TSS (CAGE cluster hg38::chrX:49264704..49264717). Transcription starting at the minor CAGE peak renders novel FOXP3 mRNA isoforms. These novel FOXP3 transcript isoforms share structural features with the canonical FOXP3 mRNA transcript (same cleavage and polyadenylation site and splicing maturation into full-length and delta2 variants), but they present a different 5´-UTR. Because there is variability in where splicing takes place, the novel transcripts can display any of two new first exons: exon -1b and exon -1c (orange arrows). **B)** Further analysis of the structure and coding potential of the novel FOXP3 transcript isoforms that emanate from the canonical promoter. Because of space constraints, we only discuss the full-length variant carrying alternative exon -1b. The canonical full-length FOXP3 mRNA isoform serves comparative purposes. The novel isoform carries a 41nt-longer 5´-UTR, harbors the complete CDS for FOXP3, and could code for the canonical proteoform (blue arrow). A potential in-frame alternative AUG start codon was identified upstream of the annotated start codon of FOXP3. Translation from this position would lead to a FOXP3 proteoform 17aa longer (4% heavier), which would be difficult to resolve using standard SDS-PAGE. A 225 aa-long uORF is predicted from a near-cognate CUG start codon (black empty arrow). The annotated FOXP3 protein and the predicted protein products (the longer FOXP3 proteoform and the 225 aa unrelated polypeptide) showed similar Kozak scores. Canon, canonical; Alt, alternative; TIS, Translation Initiation Site.

S4 Fig.
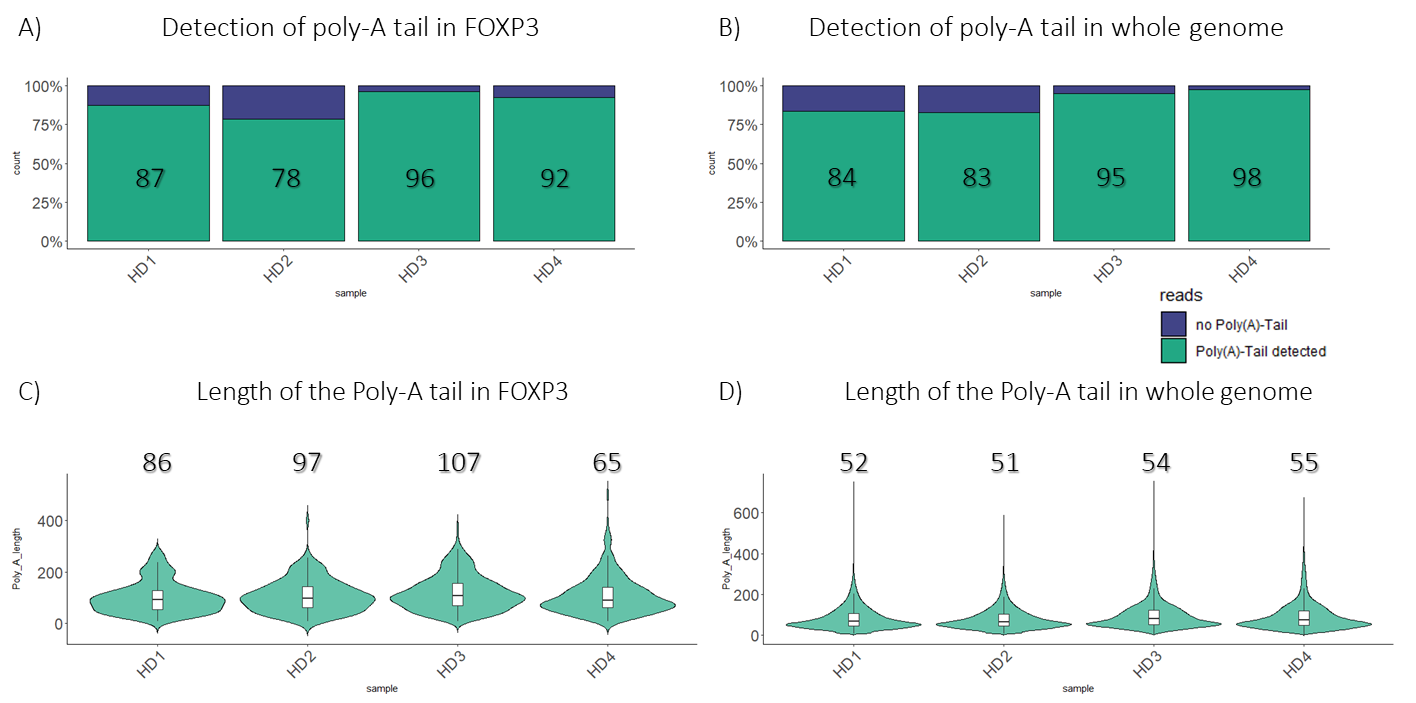


**S4 Fig. Poly(A) tail analysis of the DRS datasets.** Dorado v0.6.0 software was used to perform the poly(A) analysis. **A)** Percentage of transcripts that map to the FOXP3 locus (FOXP3 transcripts) in which a poly(A) tail was detected (green) or not (blue). **B)** Percentage of all sequenced transcripts in which a poly(A) tail was detected (green) or not (blue). On average the algorithm was able to detect poly(A) tails in 90% of the reads (range: 84% – 98%). **C)** Poly(A) tail length distribution of FOXP3 reads. **D)** Poly(A) tail length distribution of all reads. The modal length for all transcripts was on average 53nt which is within the range of modal poly(A) tail length for eukaryotes like human, mouse, *Drosophila* and *C. elegans* (50 – 100 nt). FOXP3 reads generally had longer poly(A) tails (average modal length 89nt) which could suggest greater stability (3). **(A) – (B)** The value inside the green rectangle is the percentage of transcripts in which a poly(A) was identified. **(C) – (D)** The value above the violin plots is the mode of the distribution.

S5 Fig.
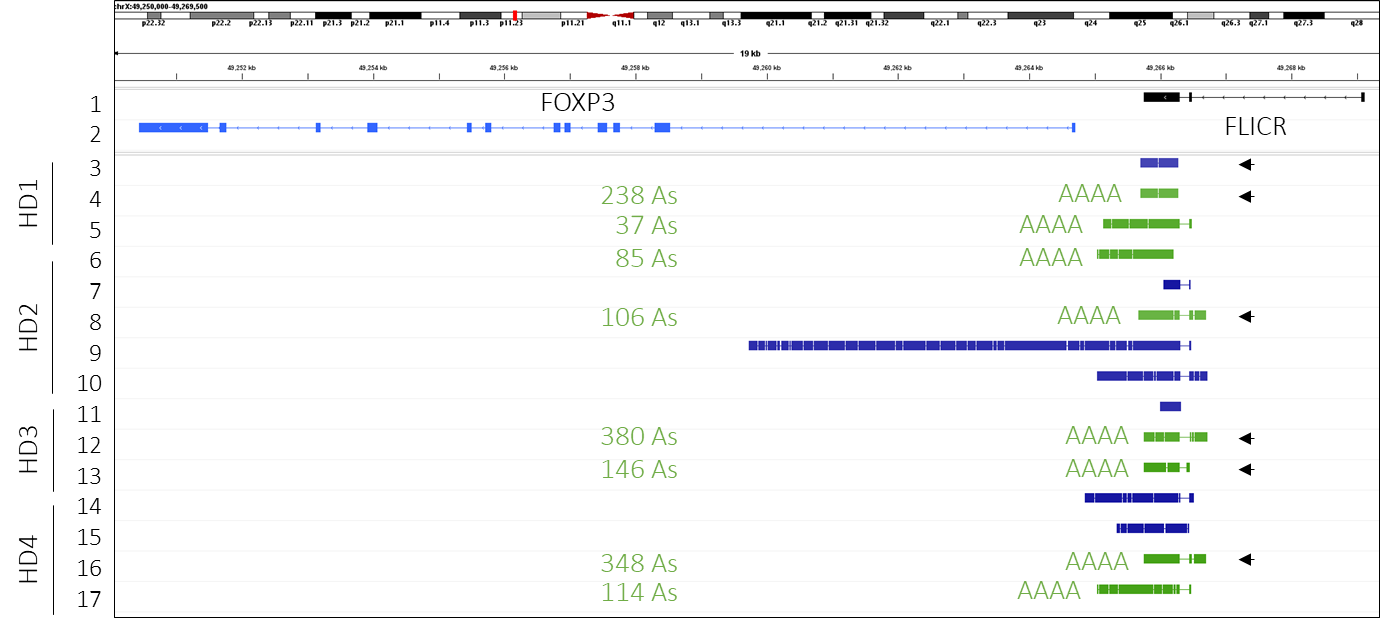


**S5 Fig. Analysis of short upstream transcripts.** IGV screenshot of the human FOXP3 locus. FLICR isoform 3 (track 1) and full-length FOXP3 mRNA (track 2) are included for genomic and structural reference. Tracks 3 – 17 include DRS reads corresponding to short upstream transcripts discussed in Fig 1A detected across four biological replicates (HD1 – HD4). We detected a poly(A) tail in only 53% (8/15) of the short upstream transcripts, with an average poly(A) tail length of 182 and a 10-fold range in length (37 – 380 As). The length of the tail is detailed to the left on the same track. Intriguingly, we could not identify classical PAS signals in the stretch between promoters. Arrowheads on the right side of the tracks point at those reads that could be interpreted as 5´ decay products of the lncRNA FLICR if we assumed that they did not emerge from the alternative promoter instead. We detected transcripts with tails as short as 10 As, suggesting that FLICR could not be missed during the poly(A)+ selection step if it had particularly short poly(A) tails. Therefore, under our experimental settings, FLICR may not be expressed above the detection level of DRS, and/or it may be more unstable than the rest of the transcripts generated from the FOXP3 locus, so that only its remnants can be detected. The former possibility is plausible as T_REGS_ cells are cultured in the presence of relatively high concentrations of IL-2, which is reported to inhibit FLICR expression (4).


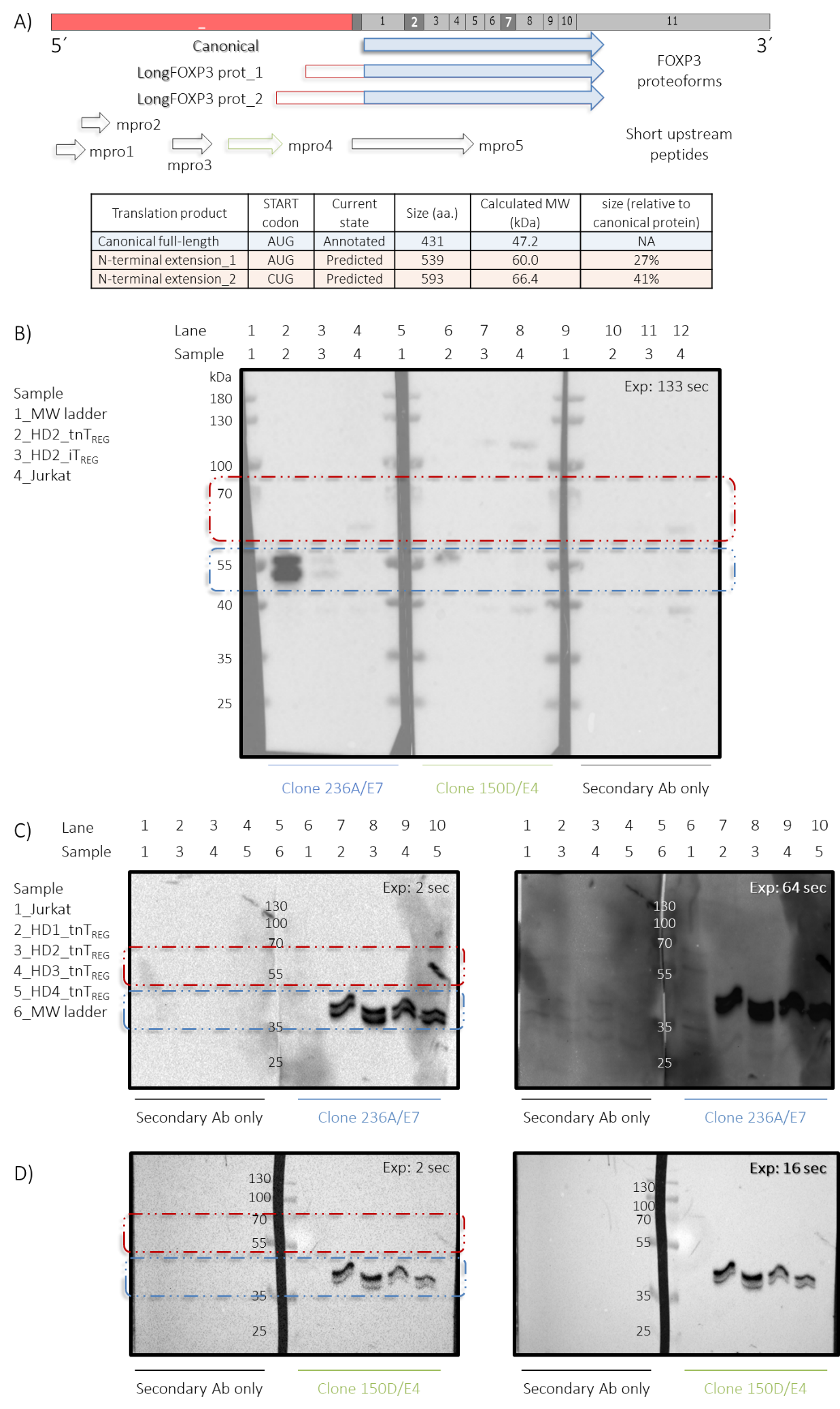
S6 Fig.

**S6 Fig. FOXP3 protein profile in Jurkat cell line and human T cell cultures using traditional western blotting**. **A)** Scheme summarizing the predicted coding capacity of longFOXP3. Inspection of the sequence confirmed the presence of the canonical coding sequence (CDS) while the existence of an AUG codon upstream of and in-frame with the annotated START codon indicated the potential existence of an N-terminally extended FOXP3 proteoform (longFOXP3 prot_1). We found that longFOXP3 could also encode another N-terminally extended FOXP3 proteoform (longFOXP3 prot_2) when considering CUG as START codon. A side-by-side comparison of both putative alternative translation initiation sites (aTISs) revealed that the near-cognate CUG codon scored better than the alternative AUG codon based on its Kozak context (0.56 vs 0.49, respectively). As a reference, the score of the canonical START codon was 0.57. Furthermore, as longFOXP3 undergoes alternative splicing of exon 2, each predicted proteoform could exist in a full-length and a delta2 variant like their canonical counterpart. **B)** Gradient (4-12%) SDS-PAGE analysis of cell lysates of tnT_REG_ and iT_REG_ cultures derived from the same donor. Under these conditions, the canonical FOXP3 protein migrated as a 55kDa protein instead of as a 47kDa protein (blue rectangle). No FOXP3-specific band was observed in the expected size range for the longFOXP3 proteoforms (red rectangle). **C)** An experiment similar to that described in **(A)**, but with four biological replicates (HD1 – HD4) analyzed with a standard 12% SDS-PAGE. Under these conditions, the canonical protein migrated as predicted based on its primary sequence (FOXP3_fl: 47.2kDa; FOXP3_∆2: 43.4kDa). Bands between 35 and 55kDa were interpreted as full-length and delta2 variants of canonical FOXP3 protein. Specific bands within the expected size range for longFOXP3 were not evident, even after over-exposure. Exposure time in upper-right corner. All samples (except 2 and 6) were run twice. After blotting and blocking, the blot was cut through the middle of Lane 5. The half to the left was incubated in primary antibody diluent, while the other half was incubated with anti-human FOXP3 monoclonal antibody 236A/E7. After washing and incubation with the HRP-labeled secondary antibody, both halves of the blot were placed back together next to each other and revealed at the same time to make the comparison as fair as possible. **D)** To corroborate that the observed bands in **C)** corresponded to canonical FOXP3, the blots were stripped and re-probed using the anti-human FOXP3 monoclonal antibody 150D/E4. The faint lower band suggests that stripping was not complete. The signal observed in line 10 inside the red rectangle is due to the tweezers used to handle the blotted membrane.

S7 Fig.
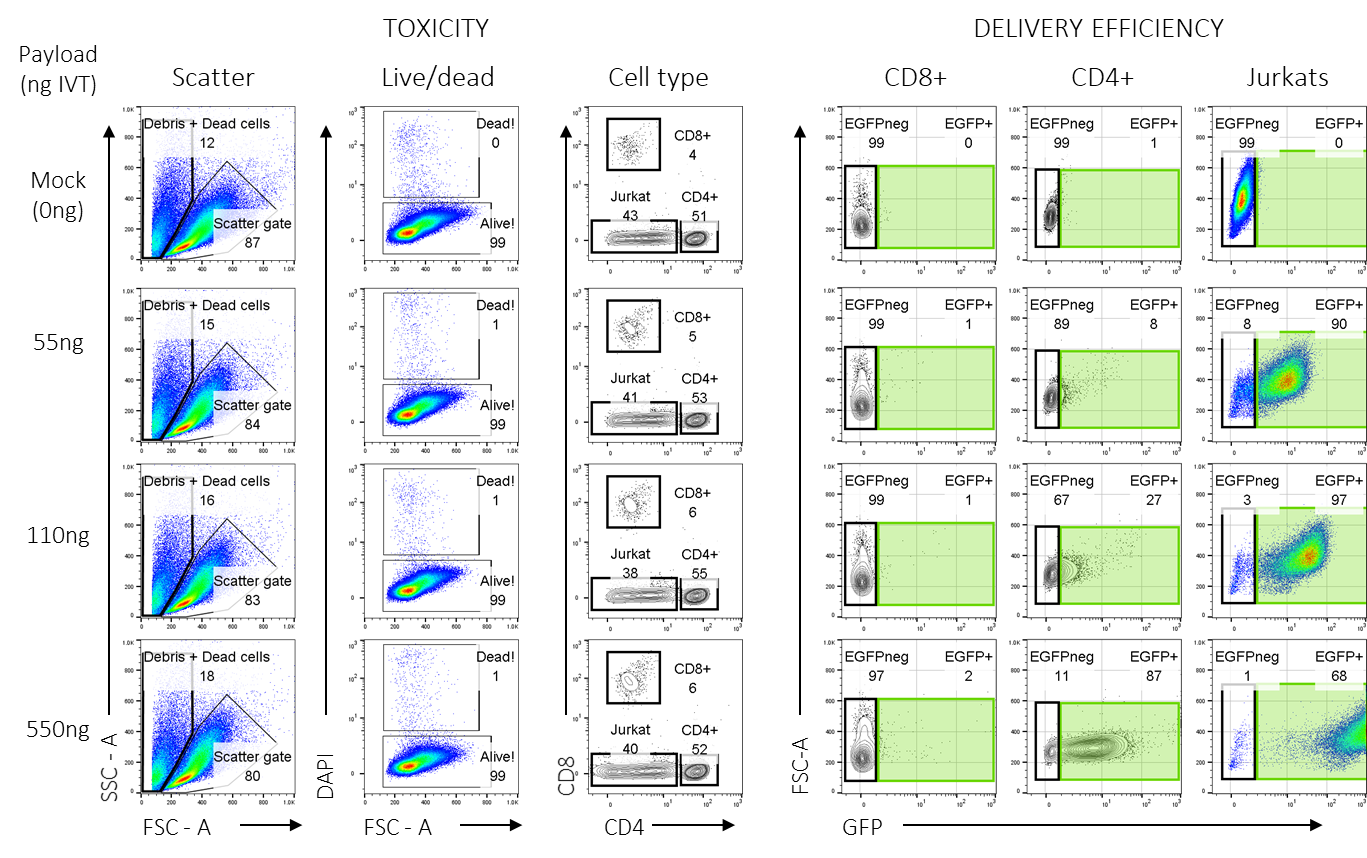


**S7 Fig. Titration of IVT RNA payload to avoid impinging on cell fitness/survival.** Cultures of primary CD4+ T cells and Jurkat cells were mixed and transfected with IVT mRNA coding for GFP, and the expression of the transgene was measured 18hs post electroporation. Four different amounts of GFP mRNA (payload) were used: 0ng (mock-transfected), 55ng, 110ng and 550ng. Two measurements were performed upon short-term co-culture: Toxicity (or cell survival) and transgene expression efficiency. CD8+ human T_CONVS_ from the same donor were added after harvesting the cells as a spike-in control to account for cell loss during staining and handling of the samples.

S8 Fig.
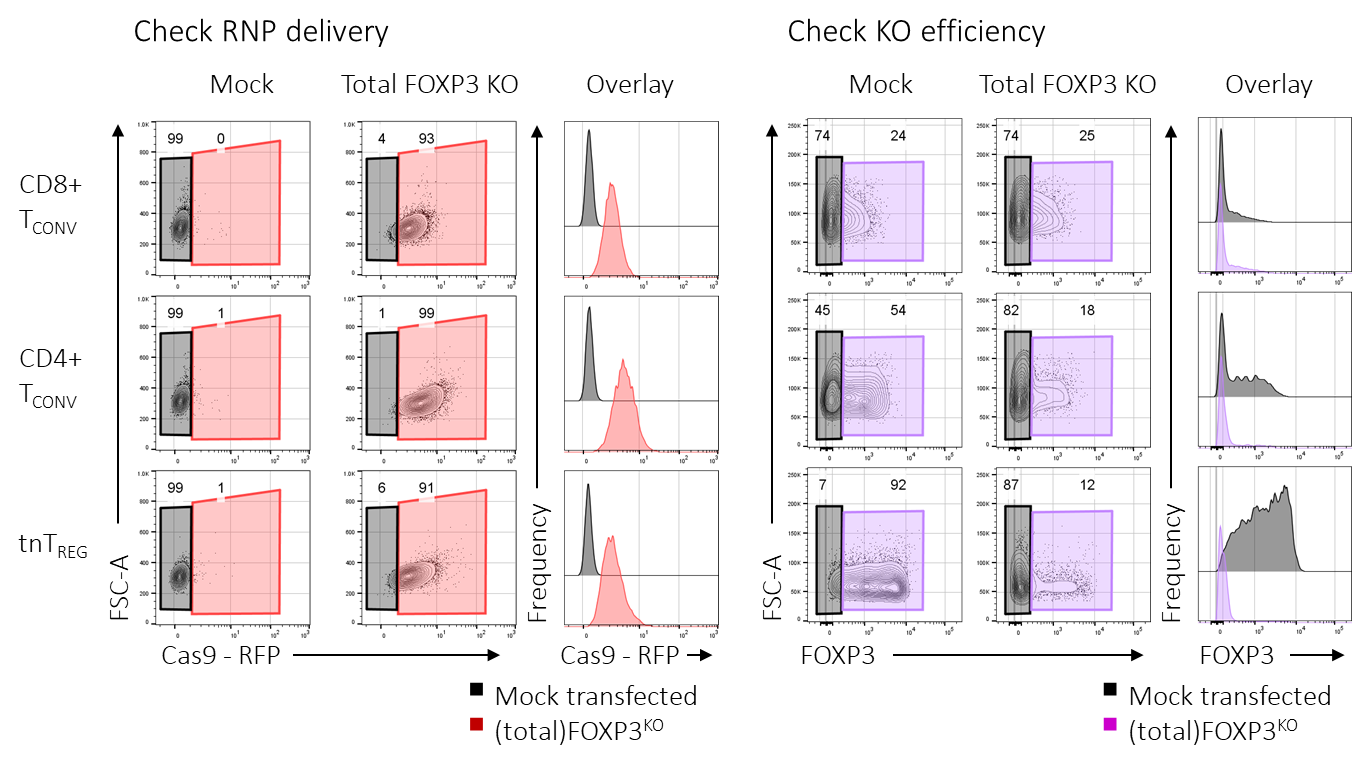


**S8 Fig. Generation of FOXP3^KO^ primary T cell lines to test the translatability of longFOXP3 into a FOXP3 proteoform.** Cultures of primary CD4+ or CD8+ human T_CONVS_ and tnT_REGS_ (from the same donor) were subjected to CRISPR-Cas9-mediated knockout of the FOXP3 gene. No gDNA sequencing was performed to assess KO efficiency. Instead, KO efficiency was assessed by FOXP3 protein levels detected by FACS analysis four days after delivery of the *in vitro*-assembled ribonucleoprotein (RNP). The recombinant (Sp)Cas9 used was a commercial fusion protein with RFP, which allowed for the assessment of RNP delivery. FACS plots to the left show RNP delivery efficiency two days after transfection. Notably, the KO efficiency in CD8+ T_CONVS_ was null, even though (a) a master mix of RNPs was used for all cell types, (b) the delivery efficiency was comparable among the three T cell subsets, and (c) the KO efficiency was efficient for the other two T cell subsets. Assuming that the mutations were introduced in the transfected CD8+ T_CONV_ cells, this could mean that FOXP3 has a (comparatively) slow turnover in CD8+ T_CONV_ cultures. Mock cells were subjected to electroporation in the absence of RNPs.

S9 Fig.
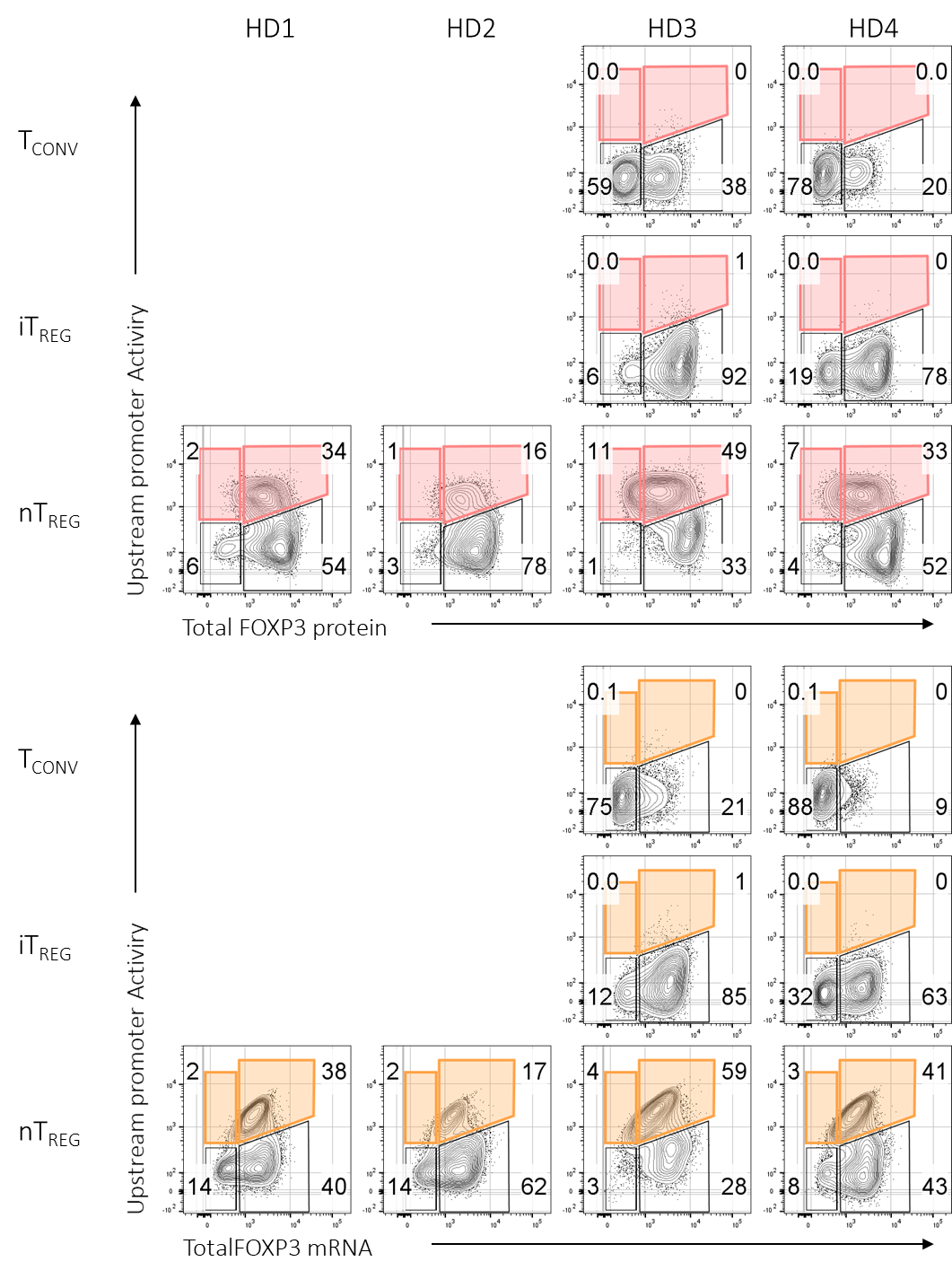


**S9 Fig. Different T cell cultures from multiple biological replicates were used to profile the activity of the upstream promoter at the single-cell level by using the PrimeFlow assay**. Instead of using standard fluorescence minus one (FMO) controls, conventional T-cells were used to set the gates. Control cultures (reactivated T_CONV_ and iT_REG_) were generated only from donors HD3 and HD4. Different samples had different levels of background in the channels assigned to the target markers, owing to the fluorophores used to tag the samples. This was particularly evident for the HD3_iT_REG_ sample with respect to the channel assigned to the UPA probe. Nevertheless, populations were clearly defined.

S10 Fig.
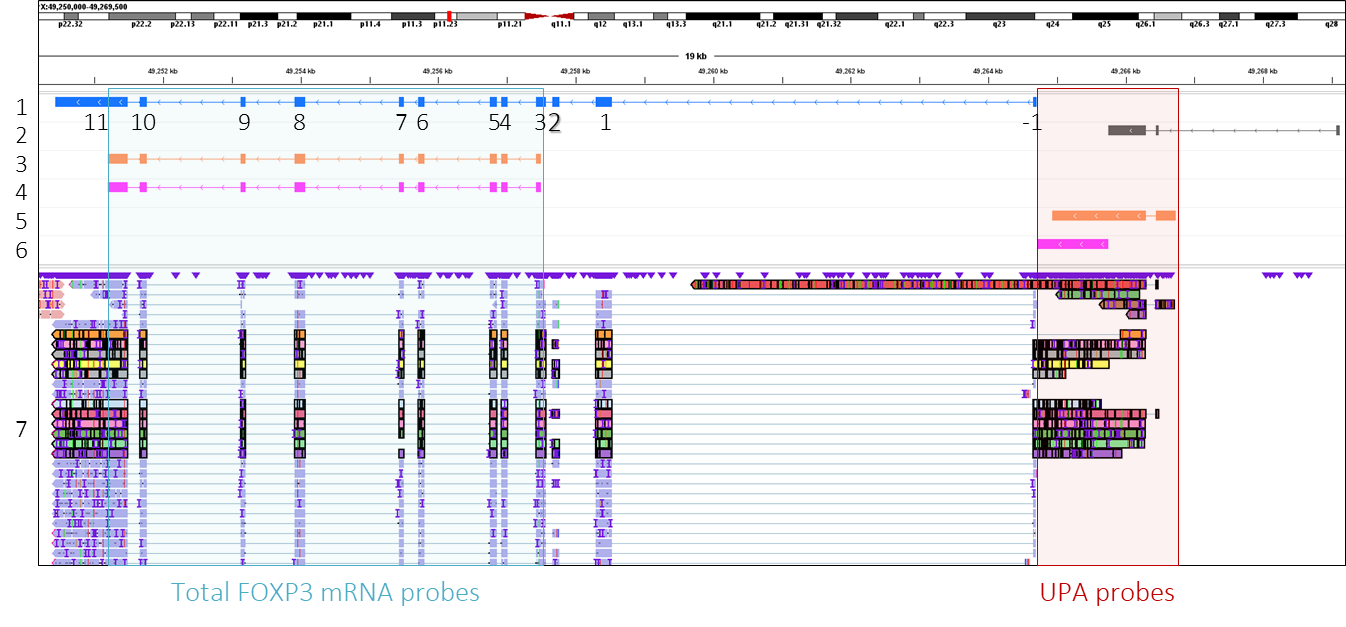


**S10 Fig. Probe design of the PrimeFlow and RNAscope assays.** IGV screenshot of the human FOXP3 locus displaying individual DRS reads aligned to the genome. Full-length FOXP3 mRNA (track 1) and FLICR isoform 3 (track 2) are included for genomic and structure reference. Each exon of FOXP3 has its given number written down below them. The probes used in the PrimeFlow assay *UPA_2* and *totalFOXP3 mRNA* are included in Tracks 3 and 5, respectively, while the probes used in the RNAscope assay *UPA_Scope* and *totalFOXP3 mRNA* are shown in tracks 4 and 6, respectively. Track 7 includes DRS data from one of the four healthy donors analyzed. The short and long transcripts emanating from the alternative promoter are highlighted in bold. Contrary to the UPA_2 probe, the *UPA_Scope* probe does not recognize any sequence of FLICR.

S11 Fig.
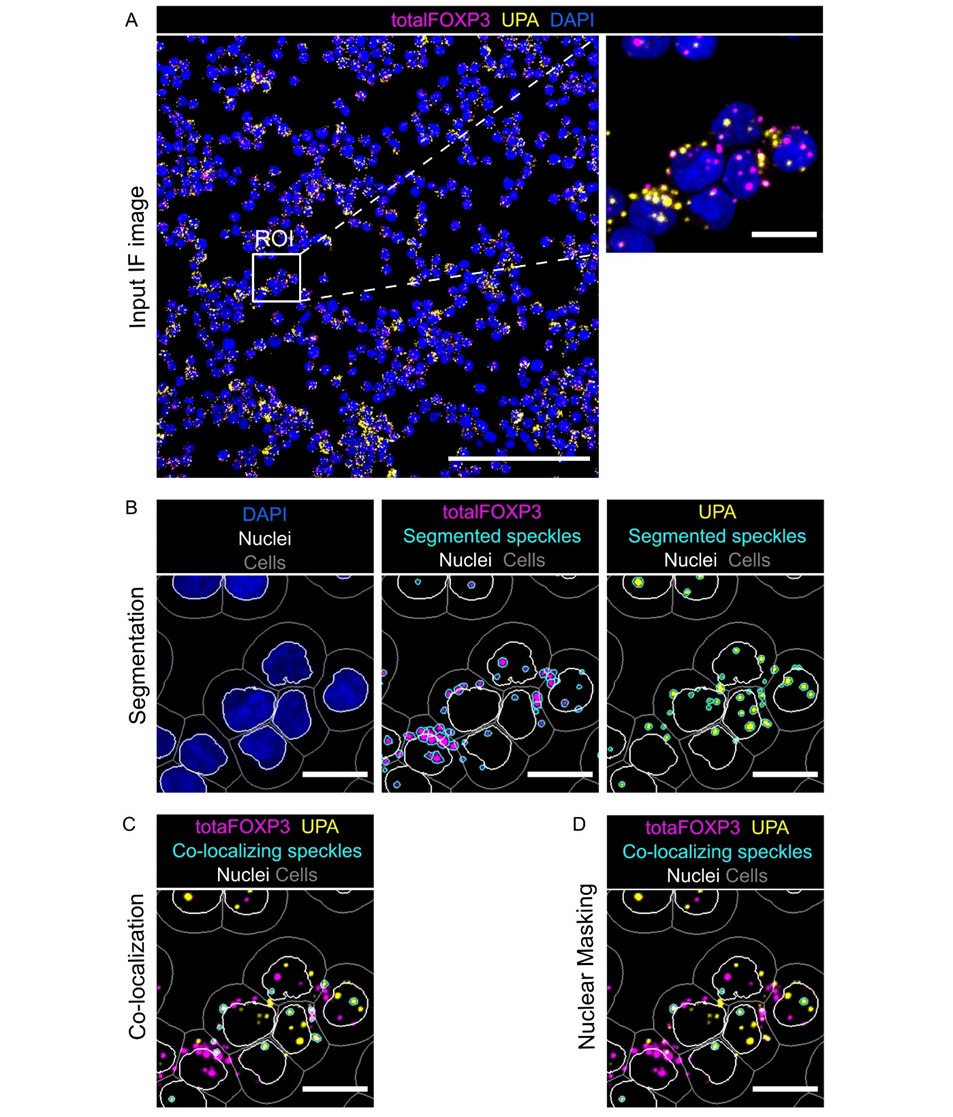


**S11 Fig. Main steps of the pipeline applied in CellProfiler to analyze the number and subcellular localization of speckles observed per cell**. **A)** Exemplary overlay of *total FOXP3 mRNA* probe (magenta), *UPA_Scope* probe (yellow), and DAPI (blue) used as input image for CellProfiler (scale bar represents 200 µm). The white square represents the zoomed-in region of interest (ROI) used to depict subsequent steps of the applied analysis strategy in the following subfigures, with a scale bar representing 10 µm. **B)** Outlines of identified nuclei (white) as primary objects and cells (grey) as expanded primary nuclei are depicted in the exemplary ROI and are overlaid with DAPI staining (left). Outlines of nuclei (white) and cells (grey), as well as identified *total FOXP3 mRNA* speckles (middle, cyan) and *UPA_Scope* speckles (right, cyan) are overlaid with RNAscope staining of *totalFOXP3 mRNA* (middle, magenta) or *UPA_Scope* (right, yellow). **C)** Result of the co-localization analysis showing outlines of co-localizing spots of *total FOXP3 mRNA* and *UPA_Scope* (cyan) together with the outlines of nuclei (white), and cells (grey), as well as RNAscope staining of *total FOXP3 mRNA* (magenta) and *UPA_Scope* (yellow) in the ROI. **D)** Results of the nuclear masking of *total FOXP3 mRNA* and *UPA_Scope* colocalizing spots are shown in the ROI. Outlines (cyan) highlight spots that show colocalizing signals of *total FOXP3 mRNA* (magenta) and *UPA_Scope* (yellow), as well as nuclear location. Additionally, outlines of nuclei (white) and cells (grey) are depicted.

S12 Fig.
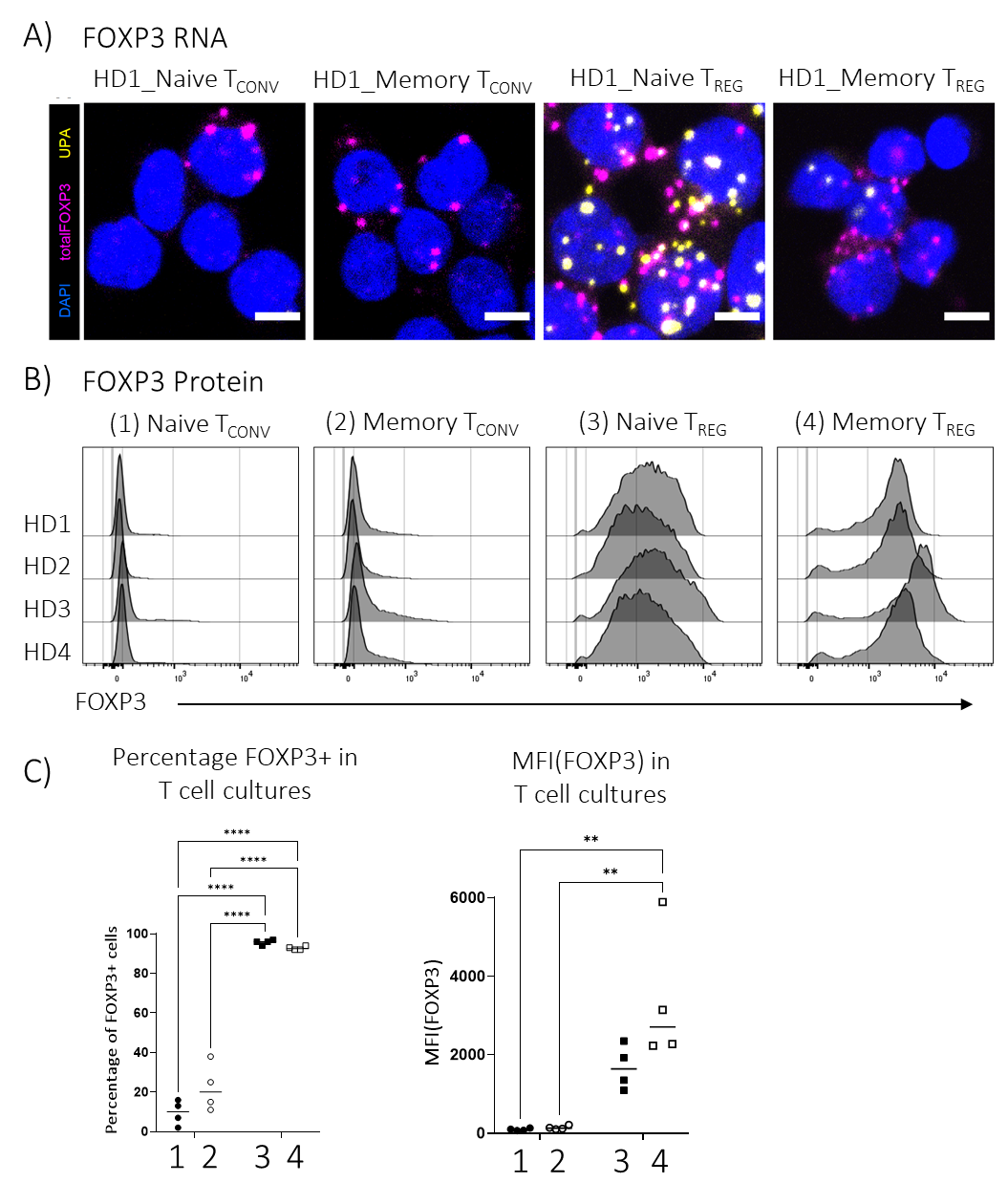


**S12 Fig. Comparison of FOXP3 RNA and protein levels with RNAscope in different T cell cultures.** **A)** Microscopy images of T cell cultures were analyzed using the RNAscope assay. Only one out of four healthy donors is presented as an example. **B)** FACS histograms of (total) FOXP3 protein levels in the four different T cell cultures analyzed for each healthy donor (HD1 – 4). **C)** Percentage of FOXP3^pos^ cells and MFI(FOXP3) in the T cell cultures used for the RNAscope assay. The inclusion of FOXP3 RNA and protein side-by-side highlights that similar to what the PrimeFlow assay showed, a high UPA signal in the T_REG_ compartment (higher percentage of UPA^pos^ cells) can be associated with a lower level of total FOXP3 staining at the single-cell level. Two-way ANOVA with Tukey’s post-hoc test. ** p<0.01, **** p<0.001. Only statistically significant differences are shown.

S13 Fig
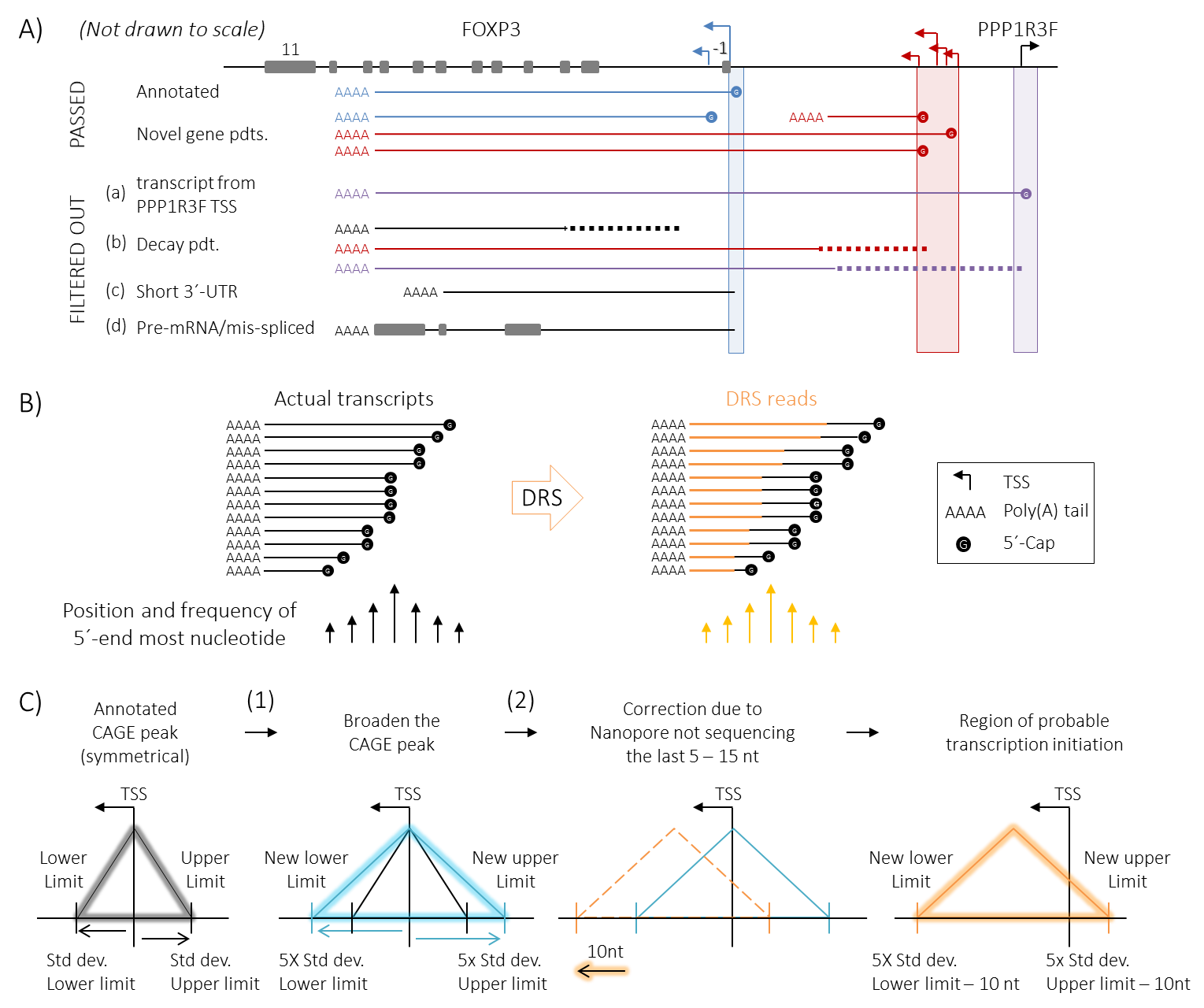


**S13 Fig. Criteria applied to select the reads used to determine the abundance of novel transcripts relative to canonical FOXP3 mRNA. A)** Scheme summarizing the criteria used for filtering DRS reads before quantification. Straight lines with a poly(A) tail and a 5`-Cap represent mature transcripts, unless otherwise mentioned in the figure. Transcripts are color-coded according to the promoter region from which they emanate (highlighted and color-coded according to S1 Fig). Dotted lines represent decay/degradation. **B)** Scheme explaining the limitation of the available DRS datasets because of the inherent limitation of the technology to retrieve the last 5 – 15 nt of the 5`-end. Even the capture of capped (“intact”) transcripts cannot render a proper profiling of the TSS. **C)** Scheme explaining the strategy used to overcome the limitation explained in **(B)**. Using public CAGE data and a series of assumptions, regions of probable transcription initiation were defined in an attempt to assign reads to a particular promoter. The average value of the reported range of uncertainty was chosen due to what it was observed for the reads that could be assigned to the main TSS within the canonical promoter.

# Supporting Tables

***S1 Table****.* ***Poly(A) tail length of canonical and longFOXP3 transcripts****. The table includes three different descriptive statistics to allow comparison of the poly(A) tails of the canonical and longFOXP3 transcripts pooled down from four biological replicates. In each case, longFOXP3 showed a higher value, but this difference was not statistically significant.*

|  | **Number of reads** | **Poly(A) length (nt)** | | |
| --- | --- | --- | --- | --- |
| **FOXP3 transcript isoform** |  | Mean | Modal | Median |
| canonical FOXP3 | 360 | 103 | 71 | 93 |
| longFOXP3 | 60 | 118 | 124 | 120 |
| Increase over canonical transcript (%) |  | 14 | 75 | 29 |

***S2 Table. Contingency table of cells with and without total FOXP3 mRNA speckles.*** Total number of cells analysed for each T cell type (condition) and number of cells that harboured totalFOXP3 mRNA speckles (W) or not (WO) are detailed.

|  | **HD45** | | | **HD46** | | | **HD49** | | | **HD50** | | |
| --- | --- | --- | --- | --- | --- | --- | --- | --- | --- | --- | --- | --- |
|  | cells | W | WO | cells | W | WO | cells | W | WO | cells | W | WO |
| Naive T_CONV_ | 875 | 175 | 700 | 825 | 163 | 662 | 1390 | 163 | 1227 | 781 | 131 | 650 |
| Memory T_CONV_ | 538 | 485 | 53 | 704 | 281 | 423 | 508 | 229 | 279 | 749 | 220 | 529 |
| Naive T_REG_ | 580 | 565 | 15 | 448 | 439 | 9 | 506 | 397 | 109 | 463 | 440 | 23 |
| Memory T_REG_ | 140 | 103 | 37 | 186 | 147 | 39 | 155 | 50 | 105 | 539 | 362 | 177 |

***S3 Table. Contingency table of cells with and without UPA_Scope speckles.*** Total number of cells analyzed for each T cell type (condition) and number of cells that harboured UPA_Scope speckles (W) or not (WO) are detailed.

|  | **HD45** | | | **HD46** | | | **HD49** | | | **HD50** | | |
| --- | --- | --- | --- | --- | --- | --- | --- | --- | --- | --- | --- | --- |
|  | cells | W | WO | cells | W | WO | cells | W | WO | cells | W | WO |
| Naive T_CONV_ | 875 | 86 | 789 | 825 | 56 | 769 | 1390 | 57 | 1333 | 781 | 37 | 744 |
| Memory T_CONV_ | 538 | 75 | 463 | 704 | 119 | 585 | 508 | 61 | 447 | 749 | 34 | 715 |
| Naive T_REG_ | 580 | 514 | 66 | 448 | 410 | 38 | 506 | 314 | 192 | 463 | 406 | 57 |
| Memory T_REG_ | 140 | 32 | 108 | 186 | 64 | 122 | 155 | 15 | 140 | 539 | 72 | 467 |

***S4 Table. Pair-wise comparison of the proportion of cells that contained at least one total FOXP3 mRNA speckle across the different T cell cultures that were analysed with the RNAscope assay****. Numbers represent p-values corrected using a logistic regression-based statistical test for contingency tables, as explained in Materials and Methods*. Extremely low values are denoted as “0.”

| **Condition** | Naive T_CONV_ | Memory T_CONV_ | Naive T_REG_ | Memory T_REG_ |
| --- | --- | --- | --- | --- |
| Naive T_CONV_ | 1 | 0 | 2.2E-67 | 5.6345E-196 |
| Memory T_CONV_ |  | 1 | 1.016E-150 | 2.052E-105 |
| Naive T_REG_ |  |  | 1 | 6.5426E-40 |
| Memory T_REG_ |  |  |  | 1 |

***S5 Table. Pair-wise comparison of the proportion of cells that contained at least one UPA_Scope speckle across the different T cell cultures that were analysed with the RNAscope assay****. Numbers represent p-values corrected using a logistic regression-based statistical test for contingency tables, as explained in Materials and Methods*. Extremely low values are denoted as “0.”

| **Condition** | Naive T_CONV_ | Memory T_CONV_ | Naive T_REG_ | Memory T_REG_ |
| --- | --- | --- | --- | --- |
| Naive T_CONV_ | 1 | 0 | 2.121E-184 | 0 |
| Memory T_CONV_ |  | 1 | 1.6515E-30 | 6.32501E-11 |
| Naive T_REG_ |  |  | 1 | 1.3255E-12 |
| Memory T_REG_ |  |  |  | 1 |

**S6 Table. List of antibodies used for FACS**.

| **Antibody** | **Fluorochrome** | **Host** | **Isotype** | **Clone** | **Company/Source** |
| --- | --- | --- | --- | --- | --- |
| CD95 | BV421 | mouse | IgG1, k | DX2 | Biolegend |
| CD4 | VioGreen | NA | Reaffinity | REA623 | Miltenyi |
| CD4 | BV510 | mouse | IgG2b, k | OKT4 | Biolegend |
| CD197 | AF488 | mouse | IgG2a, κ | G043H7 | Biolegend |
| CD8 | PerCP-Vio700 | NA | r.h. IgG1 | REA734 | Miltenyi |
| CD14 | PerCP-Vio700 | NA | r.h. IgG1 | REA599 | Miltenyi |
| CD16 | PerCP-Vio700 | NA | r.h. IgG1 | REA423 | Miltenyi |
| CD19 | PerCP-Vio700 | NA | r.h. IgG1 | REA675 | Miltenyi |
| TCRg/d | PerCP-Vio700 | mouse | IgG1κ | 11F2 | Miltenyi |
| CD25 | PE | mouse | IgG1, k | M-A251 | BioLegend |
| CD45RA | PE/Cy7 | mouse | IgG2b, k | HI-100 | Biolegend |
| CD127 | biotin | mouse | IgG1, k | A019D5 | Biolegend |
| CD8 | BV605 | mouse | IgG1, k | RPA/T8 | BioLegend |
| CD8 | BV650 | mouse | IgG1, k | RPA/T8 | BioLegend |
| CD3e | AF700 | mouse | IgG1, k | UCHT1 | Biolegend |
| Streptavidin | APC | NA | NA | NA | BioLegend |
| Streptavidin | BV421 | NA | NA | NA | BioLegend |
| Streptavidin | PE | NA | NA | NA | BD BioScience |
| CD3e | APC/Cy7 | mouse | IgG1, k | UCHT1 | Biolegend |
| CD3e | BV510 | mouse | IgG1, k | UCHT1 | Biolegend |
| CD3e | FITC | mouse | IgG1, k | UCHT1 | DRFZ in-house |
| CD3e | BUV395 | mouse | IgG1, k | UCHT1 | BD BioScience |
| CD3e | Cy5 | mouse | IgG1, k | UCHT1 | DRFZ in-house |
| CD3e | AF750 | mouse | IgG1, k | UCHT1 | DRFZ in-house |
| CD3e | BUV805 | mouse | IgG1, k | SK7 | BD BioScience |
| CD3e | BV605 | mouse | IgG1, k | SK7 | BioLegend |
| CD3e | BV711 | mouse | IgG1, k | SK7 | BioLegend |
| CD4 | AF488 | mouse | IgG1, k | TT1 | DRFZ in-house |
| CD4 | BUV395 | mouse | IgG1, k | SK3 | BD BioScience |
| CD4 | BUV737 | mouse | IgG1, k | SK3 | BD BioScience |
| CD8 | PE/Cy7 | mouse | IgG1, k | GN11/134D7 | DRFZ in-house |
| CD8 | FITC | mouse | IgG1, k | GN11/134D7 | DRFZ in-house |
| CD8 | AF647 | mouse | IgG1, k | GN11/134D7 | DRFZ in-house |
| CD8 | BV605 | mouse | IgG1, k | RPA/T8 | BioLegend |
| CD8 | BV650 | mouse | IgG1, k | RPA/T8 | BioLegend |
| CD25 | BV421 | mouse | IgG1, k | BC96 | BioLegend |
| CD25 | BV421 | mouse | IgG1, k | M-A251 | BioLegend |
| CD25 | AF647 | mouse | IgG1, k | M-A251 | BioLegend |
| CD25 | BV711 | mouse | IgG1, κ | 2A3 | BD Horizon |
| CD25 | R718 | mouse | IgG1, κ | 2A3 | BD BioScience |
| CD45 | BV421 | mouse | IgG1, k | 2D1 | BioLegend |
| CD45 | PE/Cy7 | mouse | IgG1, k | 2D1 | BioLegend |
| CD45 | AF647 | mouse | IgG1, k | 2D1 | BioLegend |
| FOXP3 | AF488 | rat | IgG2a, k | PCH101 | eBioscience |
| FOXP3 | PE | rat | IgG2a, k | PCH101 | eBioscience |
| FOXP3 | PE/Cy7 | rat | IgG2a, k | PCH101 | eBioscience |
| FOXP3 | eFluor660 | rat | IgG2a, k | PCH101 | eBioscience |
| FOXP3 | PE | mouse | IgG1, k | 259D/C7 | BioLegend |
| FOXP3 | AF647 | mouse | IgG1, k | 259D/C7 | BioLegend |
| FOXP3 | AF647 | mouse | IgG1, k | 259D/C7 | BD BioScience |
| FOXP3 | PE | mouse | IgG1, k | 236A/E7 | eBioscience |
| FOXP3 | PE | mouse | IgG1, k | 206D | BioLegend |
| FOXP1 | APC | human | r.h. IgG1 | REA682 | Miltenyi |
| Ki-67 | BV421 | mouse | IgG1, k | ki-67 | BioLegend |
| Ki-67 | PE | mouse | IgG1, k | ki-67 | BioLegend |
| Ki-67 | AF647 | mouse | IgG1, k | ki-67 | BioLegend |

**S7 Table. SgRNAs used in combination with (Sp)Cas9 to introduce mutations within the annotated CDS of FOXP3 to knockout all FOXP3 proteoforms (annotated and predicted)**. An equimolar mixture of the five sgRNAs described below was used to increase the chances of having an efficient KO.

| **sgRNA ID** | **Design tool** | **Source** | **Target site (with** PAM**)** | **NOTE** |
| --- | --- | --- | --- | --- |
| sgTotal1 | Knockout Guide Design <https://design.synthego.com/#/> | Synthego | AGGACCCGATGCCCAACCCCAGG | Predicted cut site in exon 1, downstream of the annotated START codon |
| sgTotal2 | Knockout Guide Design <https://design.synthego.com/#/> | Synthego | CCGAGGGCTTGCCAGGCCTGGGG |  |
| sgTotal3 | Knockout Guide Design <https://design.synthego.com/#/> | Synthego | CCGATGCCCAACCCCAGGCCTGG |  |
| sgTotal4 | CRISPRScan <https://www.crisprscan.org/> | CRISPRScan | AGGGCCGAGATCTTCGAGGCGGG |  |
| sgTotal5 | DESKGEN online platform [www.deskgen.com](http://www.deskgen.com) | Seki, A., & Rutz, S. (2018) (5) | CTTGAGGGAGAAGACCCCAGTGG | Predicted to cut within exon 3 |

# Supporting files

S1 file Sequencing_Merged BAM file containing all FOXP3 reads from the totality of the datasets.

S2 file Mass spectrometry_Reanalysis of public mass spectrometry data.

S3 file RNAscope_confocal microscopy pictures (.czi files).

# References

1. Gattinoni L, Lugli E, Ji Y, Pos Z, Paulos CM, Quigley MF, et al. A human memory T cell subset with stem cell-like properties. Nat Med. 2011;17(10):1290-7. Epub 20110918. doi: 10.1038/nm.2446.

2. Matos TR, Hirakawa M, Alho AC, Neleman L, Graca L, Ritz J. Maturation and Phenotypic Heterogeneity of Human CD4+ Regulatory T Cells From Birth to Adulthood and After Allogeneic Stem Cell Transplantation. Front Immunol. 2020;11:570550. Epub 20210118. doi: 10.3389/fimmu.2020.570550.

3. Passmore LA, Coller J. Roles of mRNA poly(A) tails in regulation of eukaryotic gene expression. Nat Rev Mol Cell Biol. 2022;23(2):93-106. Epub 20210930. doi: 10.1038/s41580-021-00417-y.

4. Zemmour D, Pratama A, Loughhead SM, Mathis D, Benoist C. Flicr, a long noncoding RNA, modulates Foxp3 expression and autoimmunity. Proc Natl Acad Sci U S A. 2017;114(17):E3472-E80. Epub 20170410. doi: 10.1073/pnas.1700946114.

5. Seki A, Rutz S. Optimized RNP transfection for highly efficient CRISPR/Cas9-mediated gene knockout in primary T cells. J Exp Med. 2018;215(3):985-97. Epub 20180207. doi: 10.1084/jem.20171626.
